# Obsessive–Compulsive Disorder: Comorbidity, Biomarkers, Circuits, and Treatment Strategies

**DOI:** 10.1002/mco2.71067

**Published:** 2026-11-03

**Authors:** Xiao‐Yang Zhang, Zi‐Hao Li, Li‐Ping Shen, Bing‐Qing Kong, Qi‐Peng Zhang, Ya Ke, Juan Tu, Fei Wang, Wing‐Ho Yung, Jing‐Ning Zhu

**Affiliations:** ^1^ State Key Laboratory of Pharmaceutical Biotechnology, National Resource Center for Mutant Mice, Department of Anesthesiology, Nanjing Drum Tower Hospital, Institute for Brain Sciences Department of Physiology and AI Biology, School of Life Sciences Nanjing University Nanjing China; ^2^ Chemistry and Biomedicine Innovation Center (ChemBIC) ChemBioMed Interdisciplinary Research Center Nanjing University Nanjing China; ^3^ Department of Neurosurgery Jiangnan University Medical Center Wuxi China; ^4^ School of Biomedical Sciences The Chinese University of Hong Kong Hong Kong China; ^5^ Key Laboratory of Modern Acoustics (MOE) Department of Physics Collaborative Innovation Center of Advanced Microstructure Nanjing University Nanjing China; ^6^ Early Intervention Unit Department of Psychiatry The Affiliated Brain Hospital of Nanjing Medical University Nanjing China; ^7^ Department of Neuroscience City University of Hong Kong Hong Kong China

**Keywords:** anxiety disorders, biomarkers, comorbidity, neural circuits, neuromodulation, obsessive–compulsive disorder

## Abstract

Obsessive–compulsive disorder (OCD), characterized by intrusive thoughts and repetitive behaviors, frequently co‐occurs with anxiety disorders, contributing to greater diagnostic complexity, more severe symptoms, and poorer treatment outcomes. In this review, we consider OCD in the context of its comorbidity with anxiety and examine evidence that the two conditions may share genetic, neurochemical, and circuit‐level vulnerabilities. Particular attention is given to the cortico–striato‐thalamo–cortical, frontoparietal, and frontolimbic networks, which may interact to form a self‐reinforcing cycle in which anxiety intensifies OCD symptoms, while compulsive behaviors provide transient relief but may ultimately maintain pathological patterns of brain activity. We also discuss how these shared mechanisms may shape current and emerging treatment strategies, including pharmacological approaches targeting serotonergic, glutamatergic, dopaminergic, and histaminergic systems, cognitive–behavioral interventions, and neuromodulation techniques such as deep brain stimulation, repetitive transcranial magnetic stimulation, transcranial direct current stimulation, and focused ultrasound. We further consider major depressive disorder and posttraumatic stress disorder as additional examples of psychiatric comorbidity, with overlapping neural substrates and clinical features. Taken together, these findings suggest that considering comorbidity alongside core OCD symptoms may improve our understanding of its pathophysiology, help identify clinically relevant biomarkers, and support individualized treatment, while providing a broader transdiagnostic perspective on psychiatric disorders.

## Graphical abstract

OCD has a high comorbidity with other psychiatric conditions, among which anxiety is the most frequent one. The shared genetic biomarkers and common neural circuits underlying the co‐occurrence of OCD and anxiety pave a new path for understanding the pathogenic mechanism of OCD and suggest tailored and integrative treatment therapies for OCD from the perspective of comorbidity.

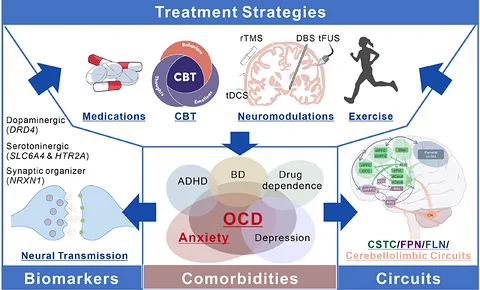

## 1 Introduction

Obsessive–compulsive disorder (OCD) is a psychiatric condition characterized by two core symptoms: obsessions and compulsions. Obsessions are persistent, intrusive, senseless thoughts, and impulses, while compulsions are repetitive, intentional behaviors, such as constantly checking, counting, or cleaning [1]. OCD significantly interferes with the individual's quality of life and ability to execute daily routine tasks. The World Health Organization has placed OCD as one of the top 10 leading causes of disability [2]. Lifetime prevalence estimates of OCD range from 1 to 3%, making it the fourth most common psychiatric disorder after those of depression, substance abuse, and social anxiety disorder (SAD) [3].

Notably, psychiatric comorbidity is common in OCD, although prevalence estimates vary substantially depending on the sampling frame (e.g., community vs. specialty‑clinic samples), diagnostic instruments, and whether lifetime or current diagnoses are assessed. For instance, in the community‑based National Comorbidity Survey Replication, up to 90% of individuals with lifetime OCD had at least one comorbid psychiatric condition [4]. Anxiety disorders are consistently the most prevalent, affecting approximately 75–80% of OCD patients in clinical and selected epidemiological cohorts, with subtype‐specific rates ranging from 24 to 70% [4, 5, 6]. Among these, generalized anxiety disorder (GAD), SAD, and panic disorder are the most frequently reported [6, 7]. Other common comorbidities include major depressive disorder (MDD), posttraumatic stress disorder (PTSD), bipolar disorder, attention‐deficit/hyperactivity disorder, and substance use disorders [4, 5]. Reflecting this substantial clinical overlap and frequent comorbidity, OCD has long been categorized as an anxiety disorder in the third and fourth editions of the Diagnostic and Statistical Manual of Mental Disorders (DSM‐III and DSM‐IV). Although DSM‐V later reclassified OCD into a distinct diagnostic category alongside related conditions such as hoarding disorder and body dysmorphic disorder [8], substantial clinical and neurobiological connections between OCD and anxiety disorders persist. Emerging evidence indicates that anxiety symptom severity not only correlates with OCD symptom intensity but also predicts poorer treatment outcomes, further underscoring an interplay rooted in shared vulnerabilities and dynamic bidirectional interactions, rather than superficial symptom co‐occurrence [9, 10, 11]. Consequently, the OCD–anxiety dyad represents a critical model for advancing the understanding of psychiatric comorbidity. Yet the mechanistic gaps underlying these interactions limit breakthroughs in treatment.

Building on epidemiological and animal studies that delineate diagnostic‐ and symptom‐level overlap, this review defines the dyad as a heterogeneous construct encompassing diagnostic comorbidity, symptom‐level overlap, and shared etiological liabilities. Synthesizing evidence across these domains, we position shared liability as a mechanistic bridge to future causal inquiry, with direct implications for diagnosis and integrated therapeutics. Accordingly, this review adopts a transdiagnostic, comorbidity‐informed perspective to systematically re‐examine OCD, integrating clinical phenomenology, genetics, neural circuitry, and treatment evidence to clarify shared mechanisms and inform personalized intervention strategies.

This review is structured to provide a coherent narrative across four main sections. First, we outline the clinical reality and prevailing conceptual models of OCD–anxiety comorbidity, emphasizing epidemiological patterns, diagnostic challenges, and limitations of current frameworks. Second, we synthesize evidence from genetic, neuroimaging, and behavioral studies to identify shared biomarkers and neural circuits, particularly within cortico–striatal–thalamo–cortical (CSTC), frontoparietal network (FPN), and frontolimbic network, that underlie their frequent co‐occurrence. Third, we evaluate established and emerging treatment strategies, including pharmacological approaches (e.g., serotonergic, glutamatergic, dopaminergic, and histaminergic agents) and neuromodulation techniques such as deep brain stimulation (DBS), repetitive transcranial magnetic stimulation (rTMS), transcranial direct current stimulation (tDCS), and focused ultrasound approaches, focusing on those that target shared mechanisms. Finally, we extend this comorbidity‐based framework to other frequently co‐occurring conditions, including MDD and PTSD, to illustrate its broader transdiagnostic utility. By integrating clinical observations with mechanistic insights and therapeutic applications, this review aims to promote a more holistic, circuit‐informed understanding of OCD and its comorbidities, ultimately contributing to the development of biomarker‐driven and personalized interventions.

## 2 The Comorbidity of OCD and Anxiety: Conceptual Models

Before examining phenotypic overlaps and interaction models, it is critical to distinguish between “anxiety” as a transdiagnostic symptom construct, encompassing excessive fear, worry, and physiological arousal, and “anxiety disorders” as formal DSM‑V diagnostic categories (e.g., GAD, SAD, panic disorder). While anxiety symptoms are ubiquitous across psychiatric conditions, this review primarily addresses the intersection of OCD with comorbid anxiety disorders. However, when investigating shared mechanisms and treatments, including animal models of anxiety‐like behaviors, we necessarily refer to “anxiety” as a dimensional construct. This distinction is crucial because the biomarkers and neural circuits discussed herein are not exclusive to categorical diagnoses. With these operational definitions in place, we first delineate how OCD and anxiety disorders converge and diverge at the phenotypic level before considering their dynamic interactions.

### 2.1 Symptom Commonalities and Differentiations

OCD has many shared symptoms with anxiety disorders. Both OCD and anxiety disorders are characterized by persistent intrusive thoughts that cause excessive fear. In addition, compulsions manifest similar avoidance behaviors associated with anxiety disorders. Despite these overlaps, the two conditions differ meaningfully in the content of intrusive thoughts and the nature of avoidance. Patients with anxiety disorders tend to worry about real‐life concerns, such as health, finances, or relationships. In contrast, individuals with OCD are obsessed with unrealistic thoughts, including aggressive, sexual, and repulsive concerns, or relatively realistic doubts about contamination, mistakes, accidents, asymmetry, and disarray [1]. Moreover, avoidance behaviors in OCD (e.g., hand washing, counting, ordering, checking), which take the form of compulsions, are highly stereotyped and repetitive, while avoidance takes various forms across anxiety disorders, such as avoiding or leaving a distressing situation, engaging in safety behaviors, and distracting oneself during a distressing situation [1, 12]. A recent study has also reported that, unlike those with OCD, patients with GAD do not exhibit active avoidance behavior, but instead have an enhanced response to Pavlovian threat cues, suggesting a more passive form of avoidance in GAD [13].

This phenotypic boundary is further refined by somatic/interoceptive OCD subtypes, wherein urinary urgency rituals serve as a critical bridge to anxiety‐related disorders [14]. While both anxiety disorders and OCD fixate on the same somatic signal, the cognitive appraisal diverges: anxiety disorders interpret the urge as impending catastrophe (“loss of control”), whereas OCD fixates on internal completeness (“just‐right” perception). This rift dictates divergent behaviors, passive avoidance (e.g., fluid restriction) in anxiety disorders versus stereotyped, goal‐directed compulsions (e.g., ritualized voiding until a precise internal sensation is met), thereby mirroring the compulsivity of classic OCD [14].

Collectively, these distinctions confirm that the observed comorbidity transcends artifactual diagnostic overlap, reflecting genuine phenotypic interactions that necessitate investigation into shared biological substrates.

### 2.2 Interaction Models of OCD and Anxiety

The comorbidity and causality between OCD and anxiety disorders constitute a classic “chicken‐or‐egg” dilemma, reflecting the complex and bidirectional nature of their interaction. Two predominant theoretical models provide distinct perspectives on the initial point of this pathological cycle. The cognitive–behavioral model of OCD has long assumed that anxiety is a result of obsessions [15, 16], contributing to compulsive behaviors attempting to alleviate anxiety and obsessional distress (Figure 1A) [17]. Alternatively, a more recent habit‐driven model (Figure 1B) suggests that compulsions in OCD, manifested as excessive habit formation, may be primary, and that anxiety and obsessions can be explained as both propagators and consequences of compulsivity [18, 19]. According to the latter model, obsessions occur as a posthoc rationalization of compulsive behaviors, which ultimately interacts with anxiety and reinforces the desire to perform compulsions through cognitive dissonance [19]. Critically, despite differing in the proposed initial component, both models converge on a fundamental insight: a self‐sustaining, vicious cycle in which maladaptive coping strategies, specifically, compulsive behaviors in the cognitive–behavioral model and cognitive rationalization in the habit‐driven model, not only fail to mitigate distress but paradoxically reinforce and exacerbate the core symptoms, trapping individuals in a persistent pathological loop.

**FIGURE 1:**
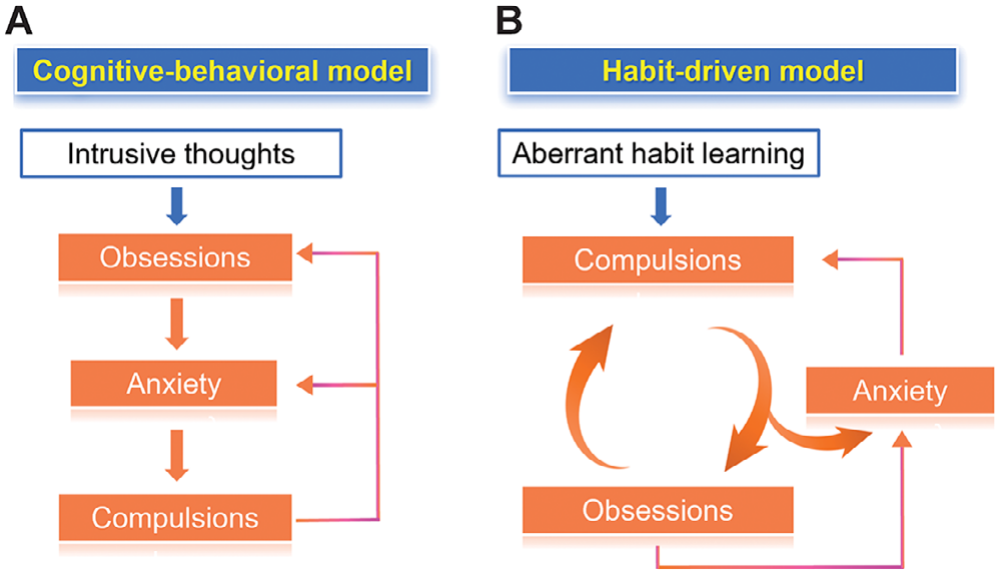
Complementary models of OCD–anxiety disorder comorbidity and bidirectional symptom cycles. (A) Cognitive–behavioral model. Intrusive thoughts are appraised as obsessions, eliciting anxiety, and motivating compulsions. Although compulsions provide transient relief, this negative reinforcement strengthens the obsession–anxiety–compulsion cycle, leading to the recurrence of intrusive thoughts. Blue downward arrows indicate the initiating cognitive appraisal, and feedback loops highlight the reinforcement mechanism. (B) Habit‐driven model. Aberrant habit learning acts as the initiating process, giving rise to compulsive urges and behaviors. Obsessions represent cognitive interpretations of these urges. Anxiety can both follow from and amplify compulsivity, further reinforcing the cycle through feedback loops. Blue downward arrows indicate the proposed initiating processes, and orange arrows indicate feedback loops in which transient relief reinforces a vicious cycle.

Growing longitudinal and clinical evidence substantiates the validity of both pathways, highlighting considerable heterogeneity in developmental trajectories. Evidence for anxiety disorders as a primary driver comes from epidemiological data showing that OCD begins at a later age than most (79.6%) comorbid anxiety disorders, especially phobias [4, 20]. Further supporting this direction, longitudinal outpatient studies reveal that clinically significant OCD symptoms frequently develop in individuals with preexisting anxiety or depressive disorders, especially following stressful or traumatic life events [21, 22]. Conversely, compelling evidence also positions OCD symptomatology as an upstream risk factor. Community‐based cohort studies with extended follow‐up periods demonstrate that subthreshold OCD symptoms can predict the subsequent onset, relapse, and persistence of both anxiety and depressive disorders [23]. Most importantly, contemporary research underscores the deeply bidirectional and interactive nature of this relationship. Longitudinal data illustrate that trait‐level anxiety vulnerabilities can predispose to and exacerbate emerging OCD symptoms, while early OCD symptoms reciprocally heighten anxiety sensitivity [11]. Moreover, structural equation modeling analyses of longitudinal data have demonstrated that both cross‐lagged and stable trait models provide a good fit, confirming that anxiety and OCD symptoms represent distinct yet dynamically interacting clusters that influence each other over time [24]. This dynamic interplay suggests that the core pathological feature may not be a fixed linear sequence, but rather an enduring, mutually reinforcing cycle.

Advancing beyond the restrictive “chicken‐or‐egg” framework requires research approaches that explicitly account for the heterogeneity within both OCD and anxiety disorder presentations. Future longitudinal studies should adopt fine‐grained, transdiagnostic subtyping, for instance, differentiating between harm‐avoidant and symmetry‐based OCD dimensions, or between GAD and panic disorder susceptibility, to clarify whether specific phenotypic constellations exhibit consistent temporal sequencing or particularly strong bidirectional coupling. This subtyping should extend beyond symptoms to include vulnerability markers, such as genetic predispositions. In parallel, mechanistically informed longitudinal designs in animal models are essential. For instance, both the *Sapap3* and *Dlgap1* knockout mouse models have demonstrated a definable anxiety‐to‐compulsion sequence within the same subject [25, 26]. Utilizing such models can experimentally test directional hypotheses: for example, whether early anxiolytic intervention can prevent compulsive onset or whether artificially induced compulsions lead to secondary anxiety. Crucially, as highlighted by research showing that modulating shared neural substrates (e.g., striatal astrocytic signaling) can normalize both compulsion and anxiety [27, 28], a key goal is to identify the common molecular and circuit mechanisms that sustain the vicious cycle. This mechanistic convergence logically points to the exploration of shared genetic biomarkers and circuits for OCD and anxiety disorders, which may reveal the common neurobiological roots of this interdependent pathology and offer novel targets for breaking the cycle.

## 3 Mechanistic Insights: Shared Biomarkers and Circuits

Building on the clinical and phenomenological overlap between OCD and anxiety disorders described above, this section examines shared neurobiological substrates of OCD and anxiety as a transdiagnostic construct. While psychological models provide a framework for understanding symptom interactions, they raise fundamental questions about the biological mechanisms underlying these relationships. This section synthesizes evidence from genetic, molecular, and systems neuroscience to delineate the common pathways that may explain the frequent co‐occurrence of these conditions. We first examine the genetic architecture and molecular mechanisms that confer shared vulnerability, drawing upon findings from familial, genomic, and multiomics studies. We then explore how these molecular‐level disruptions manifest as dysfunction in specific neural circuits, focusing on the CSTC loops and related large‐scale networks. By integrating evidence across these levels of analysis, from genes and molecules to circuits and systems, we aim to establish a coherent pathophysiological framework that bridges the gap between inherited vulnerability and the clinical expression of OCD and anxiety symptoms.

### 3.1 Shared Genetic Biomarkers

#### 3.1.1 Heritability and Familial Coaggregation

OCD is a heritable, polygenic disorder [29, 30]. Recent meta‐analytic evidence, including a 2023 synthesis of 19 family and 29 twin studies and a 2024 Swedish Twin Registry study on clinically diagnosed cases, supports a phenotypic heritability of approximately 50% [31, 32]. Notably, OCD appears to share a familial etiology with anxiety disorders. Family studies show that anxiety disorders, including GAD and agoraphobia, occur more frequently among OCD relatives, regardless of whether these relatives themselves have OCD [33]. Moreover, a strong familial aggregation and an appreciable shared genetic liability in twins have been reported between subthreshold OCD and anxiety disorders [34]. Complementing these findings, a Swedish population register‐based study, which compared intact families, families with noncohabiting biological fathers, stepfather households, and adoptive families, demonstrated that OCD is primarily transmitted across generations through genetic rather than shared environmental pathways, with rearing playing a minimal role [35]. Cross‐generationally, OCD and anxiety disorders show moderate genetic correlations, strongest between OCD and GAD, intermediate with SAD, and weakest with panic disorder [35]. Together, these findings underscore the importance of identifying shared genetic variants between OCD and anxiety disorders, as a pathway to elucidate the shared liability underlying their frequent co‐occurrence.

#### 3.1.2 Multiomics Evidence for Shared Genetic Liability

Converging evidence from genetic, epigenetic, and transcriptomic studies points to shared molecular vulnerabilities underlying the frequent co‐occurrence of OCD and anxiety disorders. These findings primarily implicate dysregulation within monoaminergic (serotonergic, dopaminergic) and glutamatergic systems, as well as in synaptic transmission and plasticity (Table 1) [36].

**TABLE 1:** Shared susceptibility genes across OCD and anxiety disorders by multiomics synthesis.

Pathway	Disorder	Genomic variation	Epigenetics	Transcriptomics
Dopamine related	OCD	*DRD4* [39]	—	*MAOB* downregulation [54]
	Panic disorder	—	*MAOA* methylation [51]	—
5‐HT related	OCD	*SLC6A4* [38] and *HTR2A* [48]	*SLC6A4* methylation [50]	*SLC6A4* and *MAOB* downregulation [54]
	Panic disorder	*SLC6A4* [45] and *HTR2A* [49]	*MAOA* methylation [51]	—
Glutamate related	OCD	*SLC1A1* [36, 41]	—	—
	Anxiety disorders	—	—	—
Synaptic structure/transmission/plasticity related	OCD	*SAPAP3* [25] and *NRXN1* [42, 43]	*OXTR* and *BDNF* methylation [50]	Synapse‐gene enrichment [55]
	Anxiety traits/GAD/SAD	*NRXN1* [42]	*USP6NL* and *CPLX1* methylation [53]	—

Abbreviations: 5‐HT, 5‐hydroxytryptamine; BDNF, brain‐derived neurotrophic factor; CPLX1, Complexin 1; DRD4, dopamine receptor D4; GAD, generalized anxiety disorder; HTR2A, 5‐hydroxytryptamine receptor 2A; MAOA/MAOB, monoamine oxidase A/B; NRXN1, Neurexin 1; OCD, obsessive–compulsive disorder; OXTR, oxytocin receptor; SAD, social anxiety disorder; SAPAP3, synapse‐associated protein 90/postsynaptic density 95‐associated protein 3; SLC1A1, solute carrier family 1 member 1; SLC6A4, solute carrier family 6 member 4; USP6NL, USP6 N‐terminal like.

In line with the fact that pleiotropic risk loci associated with psychiatric disorders are enriched for genes involved in neurotransmitter‐related pathways [37], genetic and neurobiological have suggested that genes associated with serotoninergic (*SLC6A4*) [38], dopaminergic (*DRD1*, *DRD2*, *DRD4*) [39, 40], and glutamatergic (*SLC1A1* and *SAPAP3*) [25, 41] pathways, as well as synaptic transmission and plasticity (*NRXN1*) [42, 43], are significantly involved in OCD. Large‐scale genetic correlation analyses confirm that OCD shares its strongest genetic links with anxiety disorders among psychiatric phenotypes [40]. Genome‐wide association studies (GWAS) analyses indicate significant associations within broad dopaminergic and serotonergic gene sets across stress‐related disorders, such as bipolar disorder and MDD [44]. However, GWAS approaches have yet to pinpoint specific, robust risk loci that are uniquely and reproducibly shared between OCD and individual anxiety disorders.

Candidate gene analyses provide more granular evidence for specific shared susceptibility variants. The serotonin transporter gene (*SLC6A4*), a key target of first‐line pharmacological treatments, is associated with OCD [38], panic disorder [45], and GAD [46]. *SLC6A4* variation modulates stress sensitivity, influencing both vulnerability under chronic adversity and resilience to acute extreme stress [47]. Similarly, the serotonin 2A receptor gene (*HTR2A*) shows associations with both OCD and panic disorder [48, 49]. Beyond neurotransmitter systems, the synaptic organizer gene *NRXN1* harbors risk variants and copy number variations [42] linked to both conditions, underscoring a shared neurodevelopmental susceptibility in synaptic adhesion and neural circuit formation. Collectively, these candidate genes reinforce the involvement of serotonergic and dopaminergic pathways, as well as synaptic integrity, in the comorbid etiology of OCD and anxiety.

Epigenetic studies elucidate how environmental exposures may dynamically regulate these shared genetic pathways. In OCD, differential DNA methylation has been reported in genes encoding the oxytocin receptor (*OXTR*), serotonin transporter (*SLC6A4*), and brain‐derived neurotrophic factor (*BDNF*) [50]. In panic disorder, methylation changes in genes such as *MAOA* (a key enzyme in monoamine neurotransmitter catabolism) and *CRHR1* (encoding the corticotropin‐releasing hormone receptor 1) have been linked to disease risk and treatment response [51, 52]. An epigenome‐wide association study in GAD and OCD identified differential methylation in genes including *USP6NL* (a regulator of vesicular trafficking) and *CPLX1* (encoding Complexin‐1, a key modulator of synaptic vesicle release), further linking comorbidity to synaptic function [53]. These findings position epigenetic mechanisms, particularly within serotonergic and synaptic pathways, as key modulators that may mediate the impact of environmental experiences on disease susceptibility.

Transcriptomic analyses capture the functional consequences of genetic and epigenetic variation. In peripheral blood mononuclear cells from OCD patients, significant downregulation of *SLC6A4* and *MAOB* has been observed [54]. In postmortem brain tissue, transcriptome alterations in the striatum of individuals with OCD are enriched for synapse‐associated genes, implicating the synapse as a vulnerable molecular compartment [55]. Blood‐based transcriptomic studies in SAD suggest an indirect link between early life adversity and adult pathology via immune‐related signal transduction [56]. While direct human brain transcriptomic data focusing on neurotransmitter and synaptic pathways in anxiety disorders remain scarce due to limited tissue availability, preclinical models consistently report dysregulation in serotonergic, dopaminergic, glutamatergic, and synaptic pathways [57], supporting the translational relevance of these mechanisms to anxiety pathology.

Multiomics integration reveals that the comorbidity of OCD and anxiety disorders is rooted in overlapping disruptions across several biological tiers. Shared genetic risk in monoaminergic, glutamatergic, and synaptic pathways establishes a predisposing background. Epigenetic mechanisms dynamically tune these pathways in response to environmental factors, while transcriptomic studies reflect the resultant functional state of neural systems. Together, these layers of evidence depict an interdependent, self‐reinforcing pathological cycle sustained by common molecular substrates. Future research should prioritize integrating genetic and epigenetic markers with spatially and temporally resolved single‐cell transcriptomic profiles. Such an approach will be crucial for identifying cell‐type‐ and circuit‐specific molecular signatures, thereby bridging the gap between shared genetic risk and the neural circuit mechanisms that ultimately drive comorbid pathology.

#### 3.1.3 Insights From Genetically Engineered Models

Genetically engineered animal models provide a causal framework for testing whether candidate risk genes generate OCD‐relevant compulsive phenotypes alongside anxiety‐like behaviors (Table 2) [36]. The majority of established genetic models target synaptic function, with particular emphasis on glutamatergic transmission.

**TABLE 2:** Animal models mapping OCD symptom dimensions and OCD–anxiety comorbidity‐relevant phenotypes.

Model class	Model	OCD symptom dimensions captured	OCD–anxiety comorbidity features	References
Synaptic organizer/scaffolding	*Sapap3* (*Dlgap3*) knockout mouse	*Sapap3* knockout mice show pathological repetitive grooming and other compulsive‐like repetitive behaviors, with cognitive inflexibility and aberrant habit formation.	Anxiety‐like phenotypes frequently co‐occur with compulsive‐like repetition, and early‐life social isolation can exacerbate severity.	[58, 61, 68, 69, 70]
	*Slitrk5* knockout mouse	*Slitrk5* knockout mice display compulsive‐like pathological grooming.	Anxiety‐like features have been reported alongside grooming.	[59]
	*Slitrk5a* loss‐of‐function mutant zebrafish	*Slitrk5a* loss‐of‐function zebrafish exhibit checking‐like compulsive behavior.	OCD–anxiety comorbidity features have not been established in this construct.	[60]
	*Sapap1* (*Dlgap1*) knockout mouse	*Dlgap1* knockout mice develop lesion‐inducing repetitive grooming with later developmental emergence.	Anxiety‐like avoidance can co‐occur.	[26]
Glutamate transporter	*Slc1a1* (EAAT3) overexpression in forebrain excitatory neurons (mouse)	Forebrain excitatory‐neuron *Slc1a1* overexpression increases OCD‐relevant repetitive behaviors and enhances conditioned fear.	Anxiety‐like behaviors are heightened and then reduced by chronic SSRI treatment.	[62]
	*Slc1a1* (EAAT3) loss‐of‐function mouse	Loss of EAAT3 is associated with increased stereotyped movement execution in mice.	Loss of EAAT3 is also associated with anxiety‐like behavior.	[63]
	*Slc1a1* overexpression in dopaminergic neurons (mouse)	Dopaminergic‐neuron *Slc1a1* overexpression produces stimulant‐evoked stereotypy and hyperlocomotion.	An anxiety‐like phenotype was not detected in this construct.	[64]
Developmental transcriptional regulator/microglia‐linked	*Hoxb8* mutant mouse	*Hoxb8* mutant mice show robust lesion‐inducing overgrooming.	*Hoxb8* mutants exhibit OCD‐like repetitiveness with comorbid, fluoxetine‐sensitive hyperanxiety.	[65, 66]

Abbreviations: Dlgap1/Dlgap3, discs large‐associated protein 1/3; EAAT3, excitatory amino acid transporter 3; Hoxb8, homeobox B8; OCD, obsessive–compulsive disorder; Sapap1/Sapap3, synapse‐associated protein 90/postsynaptic density 95‐associated protein 1/3; Slc1a1, solute carrier family 1 member 1; Slitrk5/slitrk5a, SLIT and NTRK‐like family member 5/5a; SSRI, selective serotonin reuptake inhibitor.

Among the most widely validated models relevant to comorbidity are synaptic organizer/scaffolding models that converge on glutamatergic dysfunction at corticostriatal synapses. In mice deficient in *Sapap3* or *Slitrk5*, both of which encode postsynaptic scaffolding proteins essential for glutamatergic synapse organization, loss of function impairs glutamatergic neurotransmission and coelicits compulsive‐like self‐grooming and anxiety‐like behaviors [58, 59]. Notably, chronic fluoxetine, a serotonergic reuptake inhibitor, alleviates overgrooming in both models and attenuates anxiety‐like behavior in *Sapap3*‐deficient mice, supporting glutamatergic dysfunction as a shared substrate and highlighting serotonergic modulation as a convergent therapeutic pathway [58, 59]. Cross‐species evidence further underscores domain specificity, as CRISPR–Cas9‐generated *slitrk5a* loss‐of‐function mutant zebrafish exhibit OCD‐like “checking” behaviors. Moreover, early‐life social isolation stress exacerbates both compulsive grooming and anxiety‐like behavior in *Sapap3*‐knockout mice, demonstrating that environmental factors can modulate comorbidity severity within a shared genetic background [61]. Similarly, *Dlgap1* (also known as *Sapap1*) knockout mice, which belong to the same *DLGAP*/*SAPAP* postsynaptic scaffold family, develop lesion‐inducing repetitive grooming along with anxiety‐like avoidance, with OCD‐like phenotypes emerging later in development, a pattern consistent with observations in *Sapap3*‐deficient models [26].

Complementing synaptic organizer/scaffolding models, perturbations of *Slc1a1*, which encodes the neuronal glutamate transporter EAAT3, provide an additional causal route to probe comorbidity‐relevant phenotypes [62]. Preclinical studies reveal a complex role: loss of EAAT3 is associated with increased stereotyped movements and anxiety in mice [63]. Forebrain excitatory‐neuron overexpression of *Slc1a1* produces OCD‐relevant repetitive behaviors together with heightened anxiety‐like behaviors, and these comorbid features are attenuated by chronic fluoxetine or clomipramine [62]. By contrast, dopaminergic‐neuron‐targeted, tetracycline‐regulated overexpression of *Slc1a1* increases amphetamine‐induced stereotypy and hyperlocomotion without detectable effects on anxiety‐like behaviors [64]. These findings indicate that the contribution of EAAT3 to OCD–anxiety phenotypes is circuit‐ and context‐specific rather than linear.

Expanding beyond the glutamatergic synapse framework, the *Hoxb8* mutant model provides important insights into how developmental regulatory genes contribute to OCD–anxiety comorbidity. Although *Hoxb8* is not among the core synaptic genes identified in human genetic studies of OCD and anxiety, it encodes a transcription factor crucial for microglial development in the brain. *Hoxb8*‐deficient mice display robust compulsive‐like overgrooming along with fluoxetine‐sensitive anxiety‐like behaviors and social impairments [65, 66]. This model thus reveals a novel mechanistic pathway through which developmental disruptions in microglia‐mediated circuit refinement can lead to the emergence of comorbid OCD‐like and anxiety‐like behaviors, in line with evidence that glial immune signaling modulates repetitive behavior [67]. The *Hoxb8* model, therefore, broadens the genetic landscape of OCD–anxiety comorbidity beyond synaptic genes to include neurodevelopmental regulators that shape neural circuit maturation.

Notably, clinical OCD exhibits substantial phenotypic heterogeneity. For example, some subtypes include urinary‐related obsessions and compulsions as well as urinary frequency and urgency symptoms [14]. Among existing models, *Sapap3*‐deficient mice recapitulate a relatively broad phenotype beyond grooming and anxiety, including impaired cognitive flexibility, aberrant habit formation, and altered valence processing [68, 69, 70]. *Sapap3*‐knockout mice also exhibit experience‐dependent alterations in grooming microstructure, accompanied by gastrointestinal dysfunction, suggesting that certain genetic models may extend to somatic and visceral symptom dimensions [71].

In summary, genetically defined animal models provide causal evidence that perturbations in neurotransmission or synaptic pathways codrive OCD‐relevant repetitive behaviors and, in a subset of constructs, anxiety‐related phenotypes. However, these models are best interpreted as domain‐specific probes of comorbidity rather than complete replicas of the full clinical syndrome spectrum.

### 3.2 Common Neural Circuits

#### 3.2.1 CSTC Circuits

The CSTC circuits have long been central to models of OCD pathophysiology [1, 72]. These parallel, partially segregated loops, originating from distinct frontal cortical regions and projecting through the striatum and thalamus, subserve affective, cognitive, and sensorimotor processing, corresponding to different OCD symptom domains [73, 74]. The affective circuit (ventromedial prefrontal cortex [vmPFC]/anterior cingulate cortex [ACC]–nucleus accumbens [NAc]–thalamus) is implicated in threat processing, including fear learning, expression, and extinction [75, 76, 77]. The cognitive circuits are further subdivided into: the dorsal cognitive circuit (dorsolateral/dorsomedial prefrontal cortex [dlPFC/dmPFC]–dorsal caudate–thalamus), which underpins executive functions like working memory, while the ventral cognitive circuit (orbitofrontal cortex [OFC]/ventrolateral prefrontal cortex [vlPFC]–ventral caudate–thalamus) is involved in response inhibition [78, 79]. Finally, the sensorimotor circuit (supplementary motor area [SMA]–putamen–thalamus) supports habit formation [80]. Notably, dimensional constructs of anxiety and compulsivity, such as excessive fear, cognitive rigidity, and stress‐facilitated habit learning, converge on dysfunction within these CSTC networks, implicating them as a neural substrate for comorbidity (Figure 2) [75, 81, 82].

**FIGURE 2:**
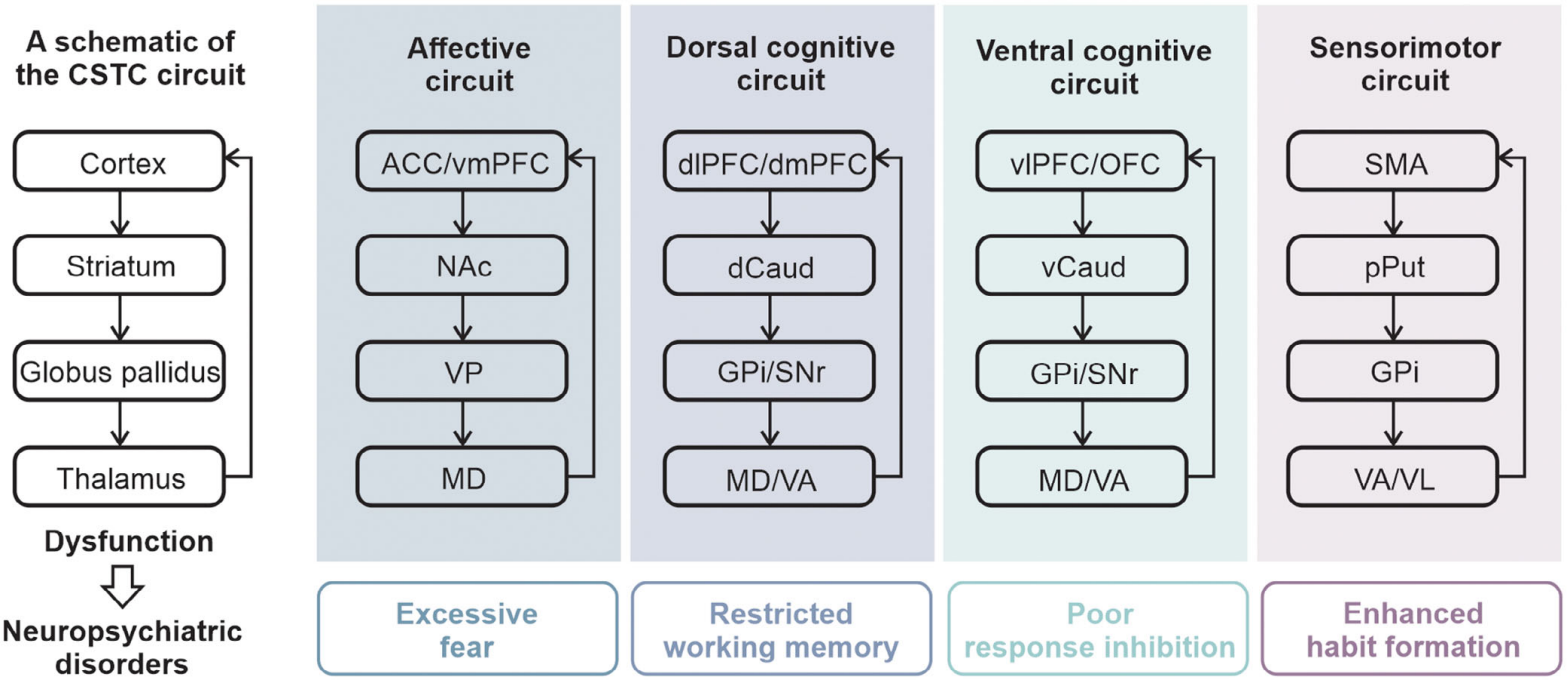
Schematic illustration of the CSTC circuits and shared neural substrates implicated in OCD and anxiety. Schematic representation of parallel, partially segregated CSTC circuits interconnecting cortical, striatal, pallidal/nigral, and thalamic nuclei. The affective loop (ACC/vmPFC–NAc–VP–MD) underlies excessive fear and threat processing. Dorsal and ventral cognitive loops (dlPFC/dmPFC–dCaud–GPi/SNr–MD/VA and vlPFC/OFC–vCaud–GPi/SNr–MD/VA) support working memory and response inhibition, respectively. The sensorimotor loop (SMA–pPut–GPi–VA/VL) governs habit formation. Dysfunction across these circuits has been consistently reported in both OCD and anxiety disorders, indicating partially overlapping circuit vulnerabilities. ACC, anterior cingulate cortex; CSTC, cortico–striatal–thalamo–cortical; dCaud, dorsal caudate nucleus; dlPFC, dorsolateral prefrontal cortex; dmPFC, dorsomedial prefrontal cortex; GPi, globus pallidus pars interna; MD, mediodorsal nucleus; NAc, nucleus accumbens; OCD, obsessive–compulsive disorder; OFC, orbitofrontal cortex; pPut, posterior putamen; SMA, supplementary motor area; SNr, substantia nigra pars reticulata; VA, ventral anterior thalamic nucleus; vCaud, ventral caudate nucleus; vlPFC, ventrolateral prefrontal cortex; VL, ventral lateral thalamic nucleus; vmPFC, ventromedial prefrontal cortex; VP, ventral pallidum.

Studies across multiple models (e.g., *Sapap3*, *Hoxb8* knockout models) confirm that corticostriatal circuit dysfunction is associated with both compulsive‐like and anxiety‐like behaviors [65, 83]. The striatum emerges as a critical hub in which interventions targeting neurons and even astrocytes can normalize comorbid phenotypes [27, 84]. The following sections synthesize evidence from human neuroimaging and animal studies, organized by the three primary CSTC subcircuits, namely, the affective, cognitive, and sensorimotor circuits, that converge on distinct striatal domains (Table 3).

**TABLE 3:** Circuit‐ and network‐level alterations across OCD and anxiety disorders.

Disorder	Affective CSTC circuit vmPFC/ACC; NAc; thalamus	Cognitive CSTC circuit dlPFC/dmPFC; OFC/vlPFC; caudate/DMS; thalamus	Sensorimotor CSTC circuit SMA; putamen/DLS; thalamus	FPN dlPFC; intraparietal sulcus; midcingulate; parietal cortex	Frontolimbic network dorsal: dlPFC/ACC, AIC; ventral: OFC, amygdala, hippocampus
OCD	Human: NAc volume and task‐evoked activation correlate positively with OCD symptom severity [86]. ACC and vmPFC show symptom‐provoked hyperactivity in OCD [88, 96]. During fear‐conditioning paradigms, vmPFC shows higher activity in OCD [91]. Mice: Compulsive severity is associated with reduced dopamine D1/D2 receptor density in NAc core [94].	Human: dmPFC and OFC show hyperactivity in OCD [88, 96]. Caudate volume is reduced in OCD [97]. Mice: Increased dmPFC–DMS projection activity suppresses compulsive grooming in *Sapap3*‐KO mice [99]. OFC activity abnormalities and reduced interneuron responses after contingency reversal impair reversal learning in *Sapap3*‐KO mice [101, 102]. DMS indirect‐pathway hyperactivity is linked to compulsive grooming; suppressing it (or fluoxetine) alleviates symptoms [104, 105].	Human: Putamen volume correlates positively with obsessive–compulsive traits [109]. Mice: *Sapap3* deletion alters DLS excitability and cholinergic modulation [112]. Rats: Quinpirole‐induced compulsive behaviors associate with structural plasticity in DLS [111].	Human: Resting‐state OCD shows reduced within‐FPN connectivity and impaired nodal integrity, particularly bilateral dlPFC [116]. EEG–fMRI indicates cross‐network dysconnectivity involving FPN, salience, and DMN in OCD [116, 117]. ERP increases within‐FPN connectivity density and decreases FPN–DMN connectivity density [118].	Human: DTI shows reduced integrity of uncinate fasciculus and frontal aslant tract in unmedicated OCD, correlating with symptom severity [130]. ENIGMA‐OCD diffusion mega‐analysis reports aberrant diffusion in fronto–thalamic and limbic‐related white‐matter pathways [131]. Lower BLA–vmPFC functional connectivity predicts better CBT response in OCD [132]. Mice: BLA–mPFC circuit is implicated in repetitive checking (quinpirole) [136].
Anxiety disorders	Human: NAc volume and task‐evoked activation correlate positively with anxiety symptom severity [87]. ACC and vmPFC show threat‐provoked hyperactivity across anxiety disorders [89, 90]. During fear‐conditioning paradigms, vmPFC activity is reduced in GAD [92]. Mice: Anxiety‐like behavior is mediated by D2‐MSNs projecting from NAc core to ventral pallidum [95].	Human: dmPFC and OFC show hyperactivity across anxiety disorders [89, 90]. Caudate volume is reduced in panic disorder [98]. Mice: dmPFC–DMS projection activity regulates anxious avoidance [100]. PV^+^ interneurons in prL and OFC are associated with basal anxiety; DMS inactivation reduces anxiety‐like behavior [103, 106]. Striatopallidal D2 receptor‐expressing neurons promote avoidance [107].	Human: Putamen volume is reduced in SAD [108]. Mice: Somatosensory cortex inputs to caudal DLS regulate anxiety in persistent pain [113]. Rats: Chronic stress facilitates DLS‐dependent habit learning [110]. DLS D1/D2 blockade increases anxiety‐like behavior [114].	Human: Across panic disorder/agoraphobia, SAD, and specific phobia, resting‐state fMRI shows diagnosis‐dependent connectivity alterations involving prefrontal, limbic, and thalamic regions, without significant symptom‐severity correlations [142].	Human: Meta‐analyses report reduced amygdala–mPFC and amygdala–ACC/dlPFC connectivity in anxiety disorders [90]. Effective‐connectivity modeling shows disrupted top‐down regulation from mPFC to amygdala in drug‐naive anxiety [133]. Mice: Chronic stress engages BLA–mPFC circuitry and induces anxiety‐like responses [137, 138].
OCD–anxiety comorbidity	Rats: PrL (vmPFC)–NAc core stimulation induces compulsive‐like and anxiety‐like behaviors; inhibition blocks stress‐induced onset [93].			Human: Task fMRI shows FPN hypoactivation during error processing and inhibitory control across OCD, SAD, and GAD [115].	Mice: *Sapap3*‐null mice show elevated ITCd Ca^2+^ activity during avoidance extinction; ITCd inhibition rescues extinction deficit [135]. Optogenetic activation of the BLA–DMS pathway induces concurrent anxiety‐like responses and compulsive grooming, and chronic activation leads to enduring compulsive phenotypes [139]. Optogenetic stimulation of *Hoxb8*‑expressing microglia in vCA1 induces both anxiety‑like and compulsive‑like behaviors [140]. Dlx5/6 manipulations in GABAergic neurons alter prelimbic and hippocampal parvalbumin‐positive neuron density alongside compulsive‐like and anxiety‐like behaviors [141].

Abbreviations: ACC, anterior cingulate cortex; AIC, anterior insular cortex; BLA, basolateral amygdala; CBT, cognitive–behavioral therapy; CSTC, cortico–striatal–thalamo–cortical; D1/D2, dopamine receptor D1/D2; dlPFC, dorsolateral prefrontal cortex; DLS, dorsolateral striatum; DMN, default mode network; dmPFC, dorsomedial prefrontal cortex; DMS, dorsomedial striatum; DTI, diffusion tensor imaging; EEG–fMRI, electroencephalography–functional magnetic resonance imaging; ENIGMA‐OCD, Enhancing Neuro Imaging Genetics through Meta Analysis‐OCD; ERP, exposure and response prevention; fMRI, functional magnetic resonance imaging; FPN, frontoparietal network; GAD, generalized anxiety disorder; Hoxb8, homeobox B8; ITCd, dorsal intercalated cell cluster; mPFC, medial prefrontal cortex; MSNs, medium spiny neurons; NAc, nucleus accumbens; OCD, obsessive–compulsive disorder; OFC, orbitofrontal cortex; PrL, prelimbic cortex; PV, parvalbumin; SAD, social anxiety disorder; Sapap3, synapse‐associated protein 90/postsynaptic density 95‐associated protein 3; SMA, supplementary motor area; vCA1, ventral CA1; vlPFC, ventrolateral prefrontal cortex; vmPFC, ventromedial prefrontal cortex.

Within the affective circuit, the NAc serves as a key node and primary target of vmPFC inputs [85]. Human functional imaging studies show that NAc volume and functional activation correlate positively with symptom severity in both OCD and anxiety disorders [86, 87]. Patients with these disorders also exhibit symptom‐ or threat‐provoked hyperactivity in the ACC and vmPFC, alongside altered vmPFC‐centered resting‐state connectivity [88, 89, 90]. However, in fear‐conditioning paradigms, the vmPFC exhibits divergent responses: hyperactivity associated with OCD versus hypoactivity associated with GAD, highlighting both shared and disorder‐specific alterations within the same circuit node [91, 92].

Experimental manipulations establish the causal role of this pathway. Optogenetic stimulation of the prelimbic cortex (PrL) subregion of vmPFC to NAc core pathway induces both anxiety‐like and compulsive‐like behaviors in rats, while inhibition of this pathway prevents their stress‐induced onset, establishing it as a mediator of comorbidity [93]. At the presynaptic level, histamine H3 heteroreceptors are expressed on glutamatergic terminals of the PrL–NAc core pathway, and their activation suppresses glutamate release, thereby alleviating comorbid anxiety and compulsive behaviors [93]. Postsynaptically, compulsive severity is associated with reduced dopamine D1 and D2 receptor density in the NAc core [94], while anxiety‐like behavior is specifically mediated by D2‐medium spiny neurons (D2‐MSNs) projecting to the ventral pallidum [95], pointing to separable postsynaptic mechanisms underlying these co‐occurring phenotypes.

In the cognitive circuit, which encompasses the dmPFC and OFC, dysfunction primarily manifests as deficits in cognitive flexibility and goal‐directed action. Human functional magnetic resonance imaging (fMRI) studies reveal hyperactivity in the dmPFC and OFC in both OCD and anxiety disorders, indicating shared cortical alterations [90, 96]. At the subcortical level, reduced volume of the caudate nucleus is consistently associated with both conditions [97, 98].

At the cortical level, increased activity of dmPFC neurons projecting to the dorsomedial striatum (DMS; the rodent homolog of the human caudate) suppresses compulsive grooming in *Sapap3*‐knockout mice [99] and controls anxious avoidance in normal mice [100]. Distinct patterns of abnormal OFC activity are linked to different OCD‐relevant phenotypes: compulsive grooming and deficits in reversal learning [101]. Mechanistically, in the *Sapap3*‐knockout model, reduced activity of OFC GABAergic interneurons in response to newly nonrewarded cues after contingency reversal leads to insufficient inhibition of OFC pyramidal neurons [102]. This results in a more pronounced reversal of response bias in D1‐MSNs toward rewarded versus nonrewarded cues, ultimately impairing reversal learning [102]. Although parvalbumin‐expressing GABAergic interneurons in the PrL and OFC are associated with basal anxiety [103], it remains to be determined whether the neuronal populations regulating cognitive inflexibility in OCD and anxiety overlap.

Converging evidence from striatal studies further supports the circuit's role in OCD–anxiety comorbidity. Hyperactivity of the striatal indirect pathway within the DMS is causally linked to compulsive grooming in *Sapap3*‐knockout mice, and suppressing this pathway, optogenetically or with fluoxetine, alleviates symptoms [104, 105]. Furthermore, DMS inactivation reduces anxiety‐like behavior [106], and striatopallidal dopamine D2 receptor‐expressing neurons in this circuit promote avoidance, a behavior relevant to anxiety disorders [107].

In the sensorimotor circuit, human neuroimaging findings reveal a more complex pattern. Putamen volume correlates positively with obsessive–compulsive traits, whereas it is reduced in SAD [108, 109]. This dissociation may reflect the circuit's differential engagement in habit formation (prominent in OCD) versus the generalized worry characteristic of anxiety disorders.

The sensorimotor loop is anchored in the dorsolateral striatum (DLS; the rodent homolog of the human putamen). Chronic stress facilitates DLS‐dependent habit learning, linking environmental risk to circuit dysfunction [110]. Quinpirole‐induced compulsive behaviors are associated with structural plasticity in DLS neurons [111], and *Sapap3*‐deletion leads to specific alterations in DLS excitability and cholinergic modulation [112]. Although a direct role for the DLS in OCD–anxiety comorbidity has not been explicitly reported, related findings suggest its involvement. For instance, inputs from the somatosensory cortex to the caudal DLS regulate comorbid anxiety in the context of persistent pain [113] and pharmacological blockade of D1 or D2 dopamine receptors in the DLS increases anxiety‐like behavior [114].

#### 3.2.2 Networks Beyond CSTC Circuits

Accumulating evidence suggests that large‐scale brain networks extending beyond the classical CSTC framework are relevant to OCD–anxiety comorbidity, particularly the FPN and frontolimbic network (Table 3).

Within the FPN, which comprises the dlPFC, intraparietal sulcus, midcingulate, and intraparietal lobe, dysfunction is consistently observed across both OCD and anxiety disorders. This network is often conceptualized as a cortical extension of the CSTC circuits, mediating higher‐order cognitive integration [115]. Multimodal electroencephalography (EEG)– fMRI studies indicate widespread dysconnectivity involving the FPN, salience network, and default mode network in individuals with OCD, emphasizing the role of abnormal cross‐network coordination in symptom manifestation [116, 117]. This dysregulation extends across diagnostic boundaries: task‐based fMRI studies in both OCD and other anxiety disorders, including SAD and GAD, consistently show hypoactivation of FPN regions during error processing and inhibitory control [115]. This common neural substrate, a functionally impaired FPN, underlies a transdiagnostic deficit in cognitive regulation.

The functional relevance of the FPN is further supported by intervention‐related neuroimaging. A longitudinal fMRI study showed that exposure and response prevention (ERP) therapy for OCD increased connectivity density within the FPN while decreasing that between the FPN and the default mode network [118]. Similarly, resting‐state fMRI studies in anxiety disorders have revealed altered FPN connectivity, with reduced connectivity associated with greater anxiety severity in GAD and disrupted interactions between the FPN and other large‐scale networks observed in SAD [119, 120]. Collectively, these findings underscore the FPN as a pivotal network whose dysregulation—both intrinsic and in interaction with other large‐scale networks—contributes to cognitive and affective disturbances common to OCD and anxiety disorders.

In the frontolimbic network, which encompasses limbic–cortical structures including the ACC, OFC, anterior insular cortex (AIC), amygdala, and hippocampus, higher‑order cognitive control is integrated with affective and interoceptive processing through dorsal and ventral pathways. The dorsal pathway, involving the dlPFC, ACC, and AIC, supports emotional regulation, interoceptive awareness, and executive control, whereas the ventral pathway, comprising the OFC, hippocampus, and amygdala, subserves reward and threat evaluation. Emerging evidence indicates that structural and functional disruptions within this network are central to both OCD and anxiety disorders.

A critical interface within this network, particularly relevant to somatic/interoceptive OCD and anxiety disorders, is the AIC. The AIC integrates bodily signals (e.g., urinary urgency, cardiac sensations) to generate subjective interoceptive awareness [121, 122]. Recent studies indicate altered insular connectivity and interoceptive processing in OCD and anxiety disorders, although the direction and subregional distribution of these abnormalities vary across tasks and diagnoses [123, 124, 125]. Crucially, downstream appraisal may diverge: in anxiety disorders, catastrophic interpretations of bodily sensations have been associated with altered amygdala and vmPFC activity, whereas sensory phenomena in OCD, including “not‐just‐right” experiences and incompleteness, have been linked more consistently to AIC, OFC, dlPFC, and sensorimotor circuitry, with ACC hyperactivity potentially reflecting heightened performance monitoring [126, 127, 128, 129]. This distinction in appraisal, catastrophizing versus incompleteness, may shape the subsequent behavioral output, channeling the shared interoceptive signal into either passive avoidance (anxiety disorders) or stereotyped compulsions (OCD).

In unmedicated OCD patients, diffusion‐tensor imaging reveals widespread reductions in white‐matter integrity within key frontolimbic network tracts, such as the uncinate fasciculus and frontal aslant tract [130]. A 2024 ENIGMA‐OCD mega‐analysis further identified aberrant diffusion in fronto–thalamic and limbic‐related white matter pathways, reinforcing the view that OCD involves distributed structural dysconnectivity across the frontolimbic network [131]. Functionally, decreased connectivity between the basolateral amygdala (BLA) and vmPFC, a core frontolimbic pathway, predicts better response to cognitive–behavioral therapy (CBT) in OCD patients, underscoring the clinical relevance of this circuit [132]. Parallel findings in anxiety disorders come from meta‑analyses of resting‑state fMRI, which consistently report altered connectivity within and between the FPN, default mode network, and limbic networks, most notably reduced connectivity in amygdala–medial prefrontal cortex (mPFC) and amygdala–ACC/dlPFC pathway [90]. Effective‐connectivity modeling in drug‐naïve GAD patients further reveals disrupted top‐down regulation from the dlPFC to the amygdala, highlighting impaired inhibitory control over right amygdala as a potential frontolimbic network mechanism in GAD [133].

Animal studies provide causal and mechanistic insights into frontolimbic network dysfunction. In Shank3 InsG3680 knock‐in mice, diminished mPFC control over BLA pyramidal neurons contributed to anxiety‐like behavior [134]. In *Sapap3*‐null mice, a failure to extinguish avoidant behavior is linked to heightened activity in the dorsal intercalated cell cluster (ITCd) of the amygdala. Optogenetic inhibition of ITCd during extinction rescues this deficit, directly implicating specific amygdala microcircuits in persistent avoidance, a phenotype relevant to OCD‐like compulsive persistence and impaired extinction processes emphasized in anxiety disorders [135]. Consistent with clinical observations, the BLA–mPFC circuit has been implicated in repetitive checking and anxiety‐like behaviors in a quinpirole‐induced OCD‐like mouse model and in acute/chronic restraint stress mouse model [136, 137, 138]. Moreover, optogenetic activation of the BLA–DMS pathway induces concurrent anxiety‐like responses and compulsive grooming, and chronic activation leads to enduring compulsive phenotypes accompanied by persistent synaptic changes, causally linking a limbic–striatal axis to comorbidity [139].

The hippocampus also plays a critical role within the frontolimbic network, particularly through its ventral sector (vCA1). Optogenetic stimulation of *Hoxb8*‑expressing microglia in vCA1 induces anxiety‑like behaviors and grooming [140]. Furthermore, studies on GABAergic neurons suggest that Dlx5/6 expression modulates both compulsive‐like and anxiety‐like behaviors [141]. Genetic manipulations altering *Dlx5/6* expression in these neurons lead to graded changes in parvalbumin neuron density in the prL and hippocampus, which are associated with the severity of compulsivity and anxiety, highlighting the role of inhibitory microcircuits in the comorbidity of these symptoms [141].

In summary, converging evidence across genetic, molecular, circuit, and network levels demonstrates that OCD and anxiety disorders arise from dysfunction within shared neural systems, particularly those regulating cognitive control, interoceptive awareness, threat evaluation, and habit formation. Collectively, these findings provide robust support for a shared liability model, explaining the high epidemiological comorbidity and establishing a mechanistic framework for probing how these substrates mediate bidirectional influences. This integrated understanding not only clarifies the pathophysiology but also provides a critical framework for translating circuit‐level insights into therapeutic innovation. Current treatments, while partially effective, often incompletely address the full comorbidity spectrum. The following sections therefore evaluate how this mechanistic knowledge can both refine existing interventions and guide the development of novel, circuit‐targeted therapies for OCD–anxiety comorbidity.

## 4 Translational Implications: From Current to Comorbidity‐Informed Therapies

Given the established shared genetic and neural circuit basis of OCD and anxiety, a key translational question arises: how can these mechanistic insights inform and refine current treatment strategies? Moreover, can they guide the development of novel, more targeted therapies? The following sections address these questions by evaluating existing and emerging interventions through the lens of comorbidity (Figure 3 and Table 4).

**FIGURE 3:**
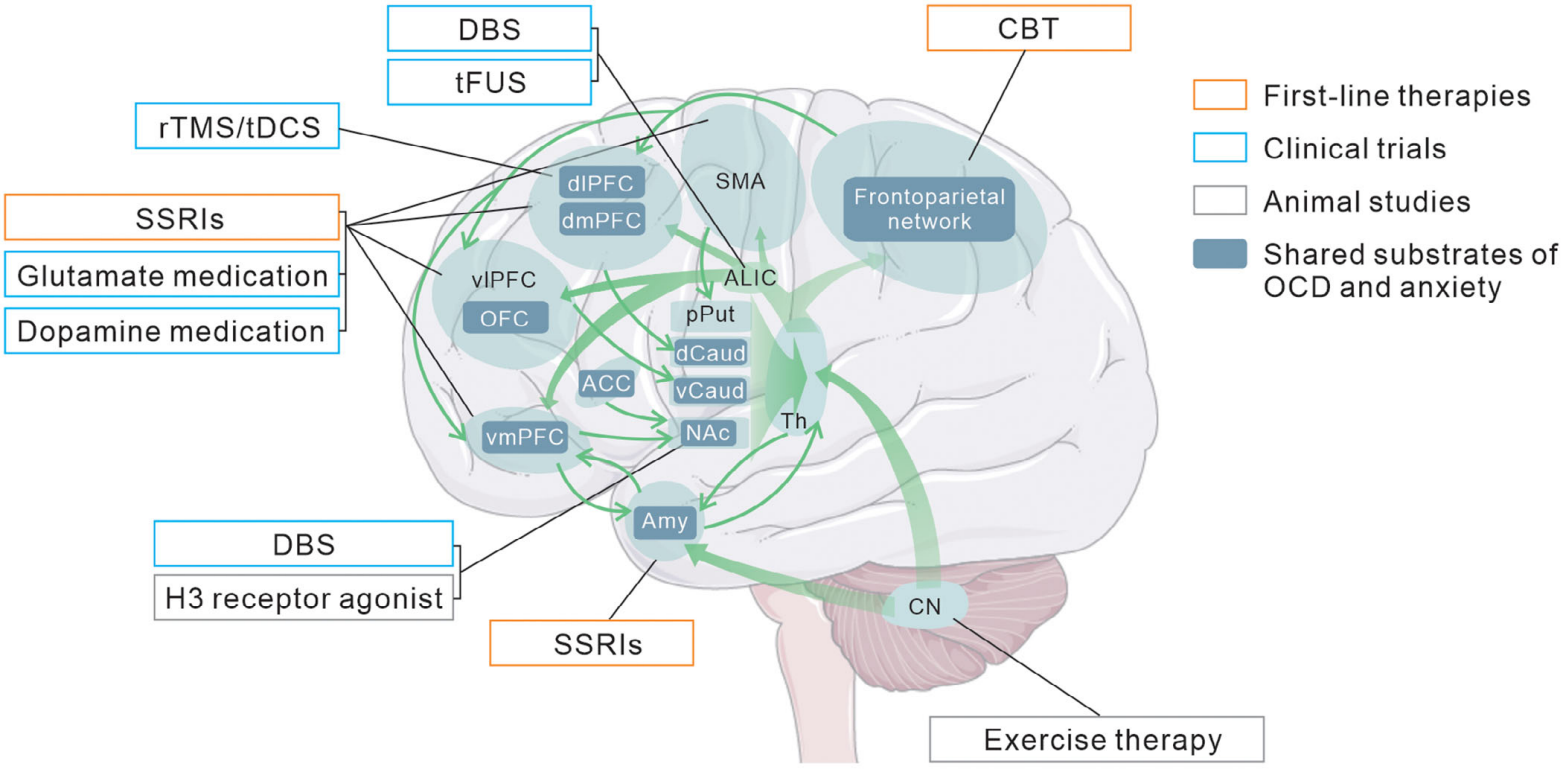
Schematic linking intervention strategies to neural circuit nodes implicated in OCD–anxiety comorbidity. Shared neural substrates are highlighted in cadet blue. Orange, blue, and gray boxes denote first‑line therapies, interventions evaluated in clinical trials, and approaches supported primarily by animal studies, respectively. Pharmacological strategies include SSRIs, which engage CSTC circuits and amygdala‑related mechanisms, and glutamatergic, dopaminergic, and histaminergic approaches (e.g., H3 receptor agonists) that modulate CSTC circuits or related limbic nodes. Cognitive–behavioral therapy (CBT) is associated with engagement and normalization of the FPN. Neuromodulation approaches include deep brain stimulation (DBS) targeting the nucleus accumbens (NAc) and/or anterior limb of the internal capsule (ALIC), repetitive transcranial magnetic stimulation/transcranial direct current stimulation (rTMS/tDCS) targeting the dorsolateral prefrontal cortex (dlPFC) or dorsomedial prefrontal cortex (dmPFC), and transcranial focused ultrasound (tFUS) targeting the ALIC. Exercise‑based interventions may alleviate anxiety via cerebello–amygdalar pathways and may modulate OCD‑related symptoms through cerebellocortical circuits. This schematic representation provides a mechanistic framework for understanding how diverse interventions converge on shared circuit disruptions, rather than a validated target map for individualized treatment selection. ACC, anterior cingulate cortex; ALIC, anterior limb of the internal capsule; Amy, amygdala; CBT, cognitive–behavioral therapy; CN, cerebellar nuclei; CSTC, cortico–striatal–thalamo–cortical; DBS, deep brain stimulation; dCaud, dorsal part of the caudate nucleus; dlPFC, dorsolateral prefrontal cortex; dmPFC, dorsomedial prefrontal cortex; H3, histamine H3 receptor; NAc, nucleus accumbens core; OCD, obsessive–compulsive disorder; OFC, orbitofrontal cortex; pPut, posterior part of putamen; rTMS, repetitive transcranial magnetic stimulation; SMA, supplementary motor area; SSRIs, selective serotonin reuptake inhibitors; tDCS, transcranial direct current stimulation; tFUS, transcranial focused ultrasound stimulation; Th, thalamus; vCaud, ventral caudate nucleus; vlPFC, ventrolateral prefrontal cortex; vmPFC, ventromedial prefrontal cortex.

**TABLE 4:** Clinical treatment evidence relevant to OCD–anxiety comorbidity.

Treatment class	Intervention	Population	Sample size	Design	Main outcomes	References
First‐line standard care	SSRIs (fluoxetine/fluvoxamine/paroxetine/sertraline/escitalopram)	Adults with primary OCD (comorbidity often allowed but rarely stratified)	Evidence base spans multiple RCTs and meta‐analyses	Mostly double‐blind, placebo‐controlled trials; acute trials typically 8–12 weeks at high dose	OCD symptoms improve, but many patients remain partial/nonresponders	[143]
	CBT/ERP	OCD; also applicable across anxiety disorders with prominent avoidance	Systematic review/meta‐analysis: 36 RCTs; *N* = 2020	Randomized trials; psychotherapy studies often use assessor‐blinded outcome ratings	Robust reduction in OCD severity; anxiety can improve when assessed but is often a secondary outcome	[158]
	Combined SSRI + CBT/ERP	OCD (often moderate–severe; comorbidity variably included)	Meta‐analysis of RCTs: 21 trials; *N* = 1113	Randomized comparisons of ERP + medication versus medication alone; follow‐up suggests better maintenance with combined treatment	Combined therapy outperforms medication alone for OCD symptoms and shows better maintenance; depressive symptoms also improve	[157]
	Internet‐delivered CBT	OCD; anxiety/depression often tracked as secondary outcomes	*N* = 101 and *N* = 120	Randomized trials, typically assessor‐blinded (single‐blind)	OCD improves; anxiety/depression can improve in parallel when measured	[161, 163]
	Inference‐based CBT (targets maladaptive reasoning)	OCD	*N* = 197	Randomized noninferiority trial; both arms active psychotherapy	Improves OCD symptoms and may aid affective symptoms; longer‐term comparative effectiveness still needs replication	[164]
Serotonergic agents beyond SSRIs	Psilocybin	OCD	*N* = 9	Modified double‐blind dose‐ranging study	Acute reductions in OCD symptoms after psilocybin; safety/tolerability characterized	[256]
	Psilocybin	Refractory OCD	Case report/case series (*N* = 1)	Uncontrolled observational reports	Marked and sometimes sustained reductions in OCD symptom burden reported; anxiety outcomes inconsistently assessed	[179, 181]
	Psilocybin	OCD	Started *N* = 19; completed *N* = 18	Blinded, fixed‐order study	Rapid reductions in compulsive symptoms lasting up to ∼1 week	[182]
	Psilocybin	Life‐threatening cancer with clinically significant anxiety/depression	*N* = 51; *N* = 29	Randomized, double‐blind, crossover designs	Substantial and durable reductions in anxiety and depressive symptoms	[184, 185]
	Psilocybin	Treatment‐resistant depression; anxiety assessed as outcome	*N* = 233	Randomised controlled, double‐blind, single‐dose trial	Improved patient‐rated anxiety following psilocybin	[180]
Glutamatergic medications	Ketamine	Treatment‐resistant OCD	Started *N* = 12; completed *N* = 10	Randomized, double‐blind, psychoactive‐controlled crossover trial	Rapid symptomatic reduction in OCD; tolerability constrained by dissociative/psychotomimetic effects	[198]
	Ketamine	OCD with suicidal ideation	Case report (*N* = 1)	Uncontrolled single‐patient report	Rapid reduction in OCD symptoms and suicidal ideation reported	[197]
	Ketamine	Treatment‐resistant OCD (subset)	Started *N* = 8; completed *N* = 5	Open‐label, 6‐week extension after acute ketamine	Symptom reductions reported; anxiety tracked via HADS‐A in TR‐OCD subgroup	[199]
	Ketamine	SAD	*N* = 18	Randomized, crossover design	Rapid and sustained anxiolytic effects	[201]
	Ketamine	Anxiety in treatment‐resistant depression	*N* = 25	Double‐blind active‐controlled crossover study	Anxiolytic effects reported	[257]
	Memantine	Trichotillomania and/or excoriation disorder (compulsive behaviors)	*N* = 100	Randomized, double‐blind, placebo‐controlled study	Reduced compulsive behaviors; implications for OCD‐spectrum symptoms	[202]
	Memantine	Anxiety disorders	*N* = 15	Single‐center, open‐label study	Preliminary evidence suggests potential benefit; not yet established for OCD–anxiety comorbidity	[203]
Dopaminergic medications	Atypical antipsychotic augmentation (agonist/antagonist)	OCD	OCD patients (*N* = 18) and controls (*N* = 18)	Randomized, double‐blind, placebo‐controlled	Treatment remediates exaggerated cingulate prediction error responses in OCD	[209]
	Tolcapone (COMT inhibitor; boosts PFC dopamine)	OCD	*N* = 20	Randomized, double‐blind, placebo‐controlled, crossover trial	Significant OCD symptom reduction reported	[207]
	Low‐dose l‐DOPA + chlorpromazine	Idiopathic dystonia patients with OCD	*N* = 26	Uncontrolled report	Striking symptomatic improvement reported; needs replication	[206]
Histaminergic medications	Hydroxyzine (H1 antagonist)	GAD	*N* = 334; 5 RCTs, *N* = 884	Multicenter randomized double‐blind placebo‐controlled trial systematic review/meta‐analysis of RCTs	Anxiety: greater HAM‐A improvement versus placebo; similar efficacy to bromazepam; meta‐analysis suggests benefit versus placebo but high risk of bias	[217, 218]
	Chlorpheniramine (H1 antagonist)	Panic disorder	Case report (*N* = 1)	Uncontrolled single‐patient report	Panic disorder: symptomatic improvement reported	[219]
	Promethazine versus lorazepam	Perioperative premedication (women undergoing anesthesia)	Promethazine: (*n* = 71); lorazepam (*n* = 67)	Double‐blind clinical trial (premedication setting)	Anxiety relief (patient‐rated) similar to lorazepam; promethazine caused dyskinetic side‐effects in six out 71	[220]
Neuromodulation and neurosurgery	DBS: subthalamic nucleus	Severe, highly refractory OCD	*N* = 16 (multicenter)	10‐month, randomized, crossover, double‐blind study (active vs. sham)	Reduced Y‐BOCS versus sham; depression/anxiety not modified; serious adverse events reported	[225]
	rTMS: supplementary motor area, low‐frequency	Medication‐resistant OCD	*N* = 21 randomized; 18 completers	Randomized, sham‐controlled, double‐blind trial	Greater Y‐BOCS reduction with active versus sham; response 67 versus 22% among completers at 4 weeks	[237]
	tDCS (add‐on) + fluoxetine	Moderate‐to‐severe OCD	*N* = 60 (30/group)	Randomized, double‐blind, sham‐controlled trial (tDCS + fluoxetine vs. sham + fluoxetine)	Y‐BOCS decreased over time, but no significant between‐group difference; tDCS was well tolerated	[238]
	MRgFUS capsulotomy (bilateral ALIC)	Medically refractory OCD	*N* = 11	Open‐label prospective study with follow‐up to 2 years	Improved OCD (Y‐BOCS) and anxiety (HAM‐A) without serious adverse effects	[242]
	MRgFUS capsulotomy long‐term follow‐up	Refractory OCD	*N* = 11 underwent capsulotomy, *N* = 10 included in 10 years of follow‐up	Long‐term observational follow‐up	Durable benefit reported (≈52% mean Y‐BOCS reduction at 10 years)	[243]

Abbreviations: ALIC, anterior limb of the internal capsule; CBT, cognitive–behavioral therapy; COMT, catechol‐O‐methyltransferase; DBS, deep brain stimulation; ERP, exposure and response prevention; GAD, generalized anxiety disorder; H1, histamine H1 receptor; HADS‐A, Hospital Anxiety and Depression Scale‐Anxiety subscale; HAM‐A, Hamilton Anxiety Rating Scale; l‐DOPA, levodopa; MRgFUS, magnetic resonance‐guided focused ultrasound; OCD, obsessive–compulsive disorder; PFC, prefrontal cortex; RCTs, randomized controlled trials; rTMS, repetitive transcranial magnetic stimulation; SAD, social anxiety disorder; SMA, supplementary motor area; SSRI, selective serotonin reuptake inhibitor; tDCS, transcranial direct current stimulation; TR‐OCD, treatment‐resistant OCD; Y‐BOCS, Yale‐Brown Obsessive Compulsive Scale.

### 4.1 Current Treatment Strategies for OCD

#### 4.1.1 Selective Serotonin Reuptake Inhibitors

Selective serotonin reuptake inhibitors (SSRIs) are currently recommended as first‐line agents for the pharmacotherapy of OCD [2], with recent international guidelines affirming their central role in clinical management [143]. SSRIs such as fluoxetine, fluvoxamine, paroxetine, sertraline, and escitalopram are typically administered at the highest tolerable doses for 8–12 weeks to assess efficacy [144, 145, 146]. The therapeutic mechanism of SSRIs is supported by both preclinical and clinical evidence. Alterations in the levels of serotonin and its metabolites in the cerebrospinal fluid of patients with OCD have been reported to be normalized by SSRI treatment [147]. Neuroanatomically, multiple subtypes of serotonin (5‐hydroxytryptamine, 5‐HT) receptors are abundantly expressed in the striatum and prefrontal cortex, providing a mechanism by which serotonergic agents can modulate CSTC circuit function (Figure 3) [148]. The inhibition of the spiking of prefrontal cortex pyramidal neurons is a powerful effect of 5‐HT, which is potentially explained by the compartmental localization of 5‐HT1A receptors on the axon initial segment [149]. Bath application of 5‐HT elicited a depolarization in fast‐spiking interneurons in the striatum via 5‐HT2C receptors [150].

Notably, SSRIs are also first‐line treatments for a wide range of anxiety disorders [151, 152]. This convergence supports the notion that SSRIs target dimensional constructs of anxiety and compulsivity via shared serotonergic pathways. It has been proposed that serotonergic modulation of the amygdala significantly contributes to the anxiolytic effect of SSRIs [153]. Therefore, CSTC circuits and the amygdala, the primary targets of the serotonergic system, are likely to be potential shared neural substrates underlying dimensional constructs of anxiety and compulsivity in both OCD and anxiety disorders. This may explain why SSRIs have become the very few antianxiety drugs that also work in treating OCD.

However, SSRI therapy in OCD patients is still unsatisfactory. At standard doses, ∼40–60% of patients show only partial or no response, and “response” in trials is commonly operationalized as a 25% reduction in Yale‐Brown Obsessive Compulsive Scale (Y‐BOCS) [146, 154, 155], highlighting the need for augmentation or alternative strategies. Augmentation with 5‑HT3 receptor antagonists has been explored to address treatment resistance [146], and some evidence supports the use of supratherapeutic SSRI dosing in partial responders [155], though this requires careful monitoring for tolerability.

Overall, while SSRIs constitute a cornerstone of OCD pharmacotherapy, their incomplete efficacy underscores the necessity of elucidating the precise causal relationship between 5‐HT dysregulation and OCD pathophysiology, as well as the exact mechanisms by which SSRIs modulate CSTC circuit function. Such insights may guide the development of more effective and personalized serotonergic interventions.

#### 4.1.2 CBT

CBT is a first‐line psychological intervention for OCD [145, 156]. It is rooted in cognitive–behavioral theories and works by breaking the OCD cycle. CBT comprises two main components, that is, cognitive reappraisal and behavioral intervention, often used together in clinical practice. The latter, specifically ERP, is one of the most essential types of CBT for OCD [157]. ERP involves purposefully increasing contact with situations that evoke anxiety symptoms, while simultaneously refraining from performing rituals, which makes OCD individuals gradually recognize their compulsions as being excessive and unreasonable [115]. By effectively recruiting cognitive control, patients can dismiss the contextually inappropriate avoidance responses that reinforce obsessions, anxiety, and the vicious cycle (Figure 1). Compulsions and rituals may thereby be alleviated or eliminated by inhibiting the desire to perform them through cognitive control. Meta‐analyses confirm that ERP yields clinically meaningful and sustained reductions in OCD symptoms [158]. Evidence supports CBT monotherapy as an effective alternative to SSRI treatment [144, 156]. Some studies further suggest that CBT monotherapy may be comparable or even superior to SSRIs in certain contexts [159]. Moreover, recent pooled data demonstrate that combining ERP with pharmacotherapy improves OCD symptoms more than pharmacotherapy alone [157].

Despite its established efficacy, CBT outcomes, compared with pharmacotherapy, rely more on practitioners’ experience and skills [160]. To mitigate variability in treatment fidelity and improve accessibility, internet‐delivered CBT (ICBT) platforms have been developed and validated. Randomized controlled trials show that ICBT achieves short‐term outcomes comparable to face‐to‐face CBT while offering superior cost‐effectiveness [161, 162, 163]. Nevertheless, there are still a large number of treatment‐refractory OCD patients who do not respond satisfactorily to adequate trials of CBT, SSRIs, or a combination of both. For these individuals, emerging CBT variants—such as inference‐based CBT, which targets maladaptive reasoning processes, and acceptance‐based ERP, which integrates mindfulness and values‐guided action—have shown encouraging, though preliminary, results in multicenter trials [164]. These approaches represent potential pathways for optimizing outcomes in treatment‐resistant populations, yet further validation is needed.

Importantly, in line with SSRI medications, CBT is also beneficial for patients with anxiety disorders. CBT interventions reduce symptoms in OCD and anxiety disorders, including GAD and panic disorder [115, 151, 156]. The mechanism may involve disruption of spiraling cycles of anxiety and avoidance. The cognitive control FPN, which exhibits significant functional imaging alterations in both OCD and non‐OCD anxiety disorders, has been considered a target for CBT [115]. Neuroimaging studies have linked successful CBT outcomes to strengthened connectivity between the dlPFC, ACC, and parietal regions [165].

However, the detailed neural mechanism underlying CBT's effect on the FPN remains obscure. Given that all of these current first‐line treatments for OCD are also effective for anxiety disorders, we hypothesize that the shared neural substrates of these two disorders may provide critical targets for the clinical treatment of OCD, and therapeutic strategies based on precise neural circuits and mechanisms underlying the comorbidity may offer promising new therapies.

### 4.2 Emerging Therapies Guided by Comorbidity Insights

#### 4.2.1 Serotonergic Drugs

Given the central role of the serotonergic system in the shared genetic and circuit‐level pathology of OCD and anxiety disorders, there is growing interest in the therapeutic potential of serotonergic agents beyond traditional SSRIs [166]. The 5‐HT2A receptor, in particular, has been identified as a common genetic biomarker for both conditions [49, 167, 168]. Clinically, untreated OCD patients exhibit a compensatory upregulation of 5‐HT2A receptor binding in the caudate nucleus [169]. Preclinical studies reveal a phenotype‐specific dissociation: reduced 5‐HT2A expression in the frontal cortex is associated with increased cognitive inflexibility in a high‐compulsive rat model [170], but with suppressed anxiety in a lipopolysaccharide‐induced mouse model [171]. Together, these human and animal findings suggest that 5‐HT2A signaling is altered in a region‐ and symptom‐domain‐specific manner in OCD and anxiety disorders, rather than showing a simple directional change. Furthermore, stress, a shared environmental risk factor for OCD and anxiety, modulates serotonergic signaling across key nodes of the CSTC, FPN, and frontolimbic network. For instance, maltreated nonhuman primates exhibit significantly lower 5‐HT1A receptor binding in prefrontal and limbic regions, along with reduced 5‐HT2A receptor binding in striatal and prefrontal regions [172]. Collectively, these observations suggest that 5‐HT receptor signaling, particularly involving the 5‐HT2A subtype, is implicated in the pathophysiology of both conditions, warranting further investigation as a potential intervention target for their comorbidity.

Preclinical models offer important mechanistic insights. In the *Sapap3*‐knockout mouse model of compulsive behavior, acute administration of psilocybin, a high‐affinity agonist at 5‐HT1A, 5‐HT2A, and 5‐HT2C receptors, produces a durable reduction in excessive self‐grooming [173], an effect also observed with psychedelic mushroom extract [68]. Interestingly, while psilocybin did not alter anxiety‐like behaviors in this specific model [173], it attenuated anxiety‐like behaviors in a chronic unpredictable mild stress paradigm [174]. Studies focusing on the 5‐HT2A receptor, a primary target, show that its activation suppresses fear expression via actions in the amygdala [175]. Nevertheless, the attenuation of compulsive marble‐burying by psilocybin in mice is not blocked by 5‐HT2A or 5‐HT1A antagonists [176], pointing to the involvement of additional mechanisms. Emerging evidence suggests that the 5‐HT1B receptor may contribute to some of the longer‐lasting behavioral effects of psilocybin, particularly in stress‐related contexts [177].

Psilocybin and its active metabolite psilocin have attracted considerable clinical attention [178]. Early‐phase studies, though limited in scale and design, report promising effects on both OCD and anxiety [179, 180]. Case reports describe marked and sustained symptomatic improvement in patients with refractory OCD following psilocybin use [179, 181]. Small open‐label and pilot trials further indicate that a single 10 mg dose of psilocybin is well‐tolerated and can lead to rapid, clinically meaningful reductions in compulsive symptoms lasting up to 1 week [182]. In addition, a randomized, double‐blind study demonstrated that psilocybin could be safely administered to individuals with OCD and was associated with acute reductions in core symptoms [183]. Beyond OCD, more rigorous trials support psilocybin's anxiolytic potential. A Phase 2 double‐blind trial in treatment‐resistant depression reported significant improvements in patient‐rated anxiety following psilocybin administration [180], and randomized controlled trials in patients with life‐threatening cancer have documented substantial and durable antianxiety and antidepressant effects [184, 185].

#### 4.2.2 Glutamatergic Medications

Genetic and preclinical evidence firmly implicates glutamatergic dysfunction in the shared pathophysiology of OCD and anxiety. As highlighted in Section 3.1.3, *Sapap3*‐, *Slitrk5*‐, *Sapap1*‐, and *Slc1a1‐*knockout mice, which disrupt corticostriatal glutamate transmission, concurrently exhibit compulsive‐like grooming and anxiety‐like behaviors [26, 58, 59, 61, 63]. Moreover, human neuroimaging studies using proton magnetic resonance spectroscopy consistently report altered glutamate and glutamate–glutamine levels, often alongside GABA abnormalities, within frontal–striatal nodes of the CSTC circuits in individuals with OCD. These neurochemical imbalances in regions like the ACC and SMA correlate with symptom severity and compulsive behaviors, providing a direct neurochemical link to the disorder [186]. Furthermore, such glutamate–GABA disturbances represent a transdiagnostic neurochemical signature of anxiety and compulsivity, commonly observed across OCD and anxiety disorders [186, 187]. Supporting a causal link, recent optogenetic studies in rodents have pinpointed specific glutamatergic pathways, such as projections from the PrL to the NAc core, that simultaneously mediate both anxiety‐ and compulsion‐like behaviors [93].

Preclinical studies have leveraged these genetic and circuit models to explore therapeutic targets and establish causal pharmacology. Research in *Sapap3*‐knockout mice indicates that repetitive behaviors are driven, in part, by increased signaling through metabotropic glutamate receptor 5 (mGluR5) in the striatum [188, 189]. Consequently, mGluR5 negative allosteric modulators (e.g., MTEP) effectively reduce both compulsive‐like grooming and anxiety‐like behaviors in this model [190]. Furthermore, mGluR2/3 agonists and positive allosteric modulators (e.g., LY404039, AZD‐8529) produce dose‐dependent reductions in compulsive checking behaviors in rodent paradigms [191]. The role of ionotropic receptors is also evident, as competitive N‐methyl‐d‐aspartate receptor (NMDAR) antagonists (e.g., NVP‐AAM077, Ro25‐6981) significantly inhibit anxiety‐like and compulsive behavior in a quinpirole‐induced OCD model [192]. However, pharmacological effects can be model‐dependent; for instance, acute ketamine administration did not reduce anxiety or grooming in the *Sapap3*‐knockout model, and memantine was found to impair adaptive anxiety responses in mice, highlighting the complexity of NMDAR modulation [193, 194].

Clinically, this strong preclinical rationale has spurred the investigation of glutamatergic modulators as augmentation strategies for treatment‐resistant OCD and anxiety disorders [195, 196]. Ketamine has shown rapid reductions in OCD symptoms and suicidal ideation in case reports and a randomized crossover trial, with sustained benefits observed during open‐label extension [197, 198, 199]. Preliminary evidence also supports its potential in adolescent OCD, although psychomimetic effects require careful clinical monitoring [200]. Its rapid and sustained anxiolytic effects have also been confirmed in SAD [201] and treatment‐resistant anxiety [195]. Memantine augmentation has yielded statistically significant benefits in reducing compulsive behaviors, such as hair‐pulling and skin‐picking, in randomized controlled trials [202] and is being explored for anxiety disorders [203].

#### 4.2.3 Dopaminergic Medications

Alterations in genes such as *DRD4*, *MAOA*, and *MAOB* point to a shared dopaminergic vulnerability in OCD and anxiety disorders. Complementing this, circuit‐level research within the CSTC framework provides causal evidence for dopamine's involvement. For instance, NAc dopamine depletion impairs strategy‐switching flexibility [204], a dimensional impairment that often occurs in both OCD and anxiety disorders. Chemogenetic activation of dopaminergic neurons projecting to the DMS induces fronto–cortical hypoactivity and attenuates rat anxiety and compulsive behaviors [205].

While dopamine antagonists (e.g., atypical antipsychotics) remain the best‐established augmenting agents for treatment‐resistant OCD, their utility is constrained by variable efficacy and significant side‐effect burdens [154]. This has spurred interest in prodopaminergic strategies. Promisingly, a small exploratory clinical study indicates that low‐dose l‐DOPA combined with chlorpromazine can yield striking benefits in OCD [206]. Further supporting this approach, a double‐blind, placebo‐controlled trial of tolcapone—a catechol‐O‐methyltransferase (COMT) inhibitor that enhances prefrontal dopamine—demonstrated significant symptom reduction in OCD [207]. Although direct evidence in anxiety disorders remains sparse, preliminary open‐label findings in affective disorders suggest potential therapeutic effects of tolcapone, warranting further investigation of its relevance to anxiety‐related symptoms [208]. Neuroimaging studies provide a plausible mechanistic basis for this apparent therapeutic paradox: both dopaminergic agonists and antagonists have been shown to normalize aberrant error‐signaling in the ACC [209], a neural correlate shared by OCD and anxiety symptomatology.

#### 4.2.4 Histaminergic Medications

The central histaminergic system, primarily synthesized in the hypothalamic tuberomammillary nucleus (TMN), is emerging as a key neuromodulator within neural substrates shared by OCD and anxiety disorders, particularly the NAc and amygdala [93, 210]. Within the NAc core, histaminergic projections from the TMN act on presynaptic H3 receptors to selectively inhibit glutamatergic transmission [93, 211], and stress‐evoked histamine signaling in this region induces long‐term depression of excitatory inputs to dopamine D1‐MSNs [211]. Additionally, histamine modulates H1, H2, and H3 receptors in the NAc shell, where it regulates parvalbumin‐positive and cholinergic interneurons in a synapse‐ and cell type‐specific manner, bidirectionally shaping local microcircuit activity [212]. Functionally, microinjection of the H3 selective agonist (R)‐α‐methylhistamine (RAMH) into the NAc core significantly reduces stress‐induced anxiety‐like and obsessive–compulsive‐like behaviors in rats [93], and optogenetic activation of the TMN–NAc histaminergic pathway suppresses the PrL‐to‐NAc glutamatergic projection, producing similar behavioral effects [93]. Parallel work has shown that TMN‐derived histaminergic inputs to the lateral subdivision of the central nucleus of the amygdala inhibit glutamatergic transmission via H3 receptors, pointing to the amygdala as another shared node of histaminergic regulation [210].

Beyond these shared circuits, separate lines of evidence support histamine's involvement in OCD‐ and anxiety‐related behaviors. *Hdc*‐knockout mice, which model tic/OCD pathophysiology due to a lack of histamine synthesis, exhibit both white matter abnormalities reminiscent of clinical OCD and compulsive‐like behaviors [213]. Moreover, enhanced central histaminergic transmission, achieved via administration of histamine, an H3 antagonist, or the precursor l‐histidine, reduces compulsive‐like behaviors [214]. Separately, studies in anxiety models have identified a histaminergic pathway from the TMN to the bed nucleus of the stria terminalis that modulates anxiety‐like behavior via postsynaptic H3 receptors on GABAergic neurons, expanding the known circuitry involved in anxiety regulation [215]. Collectively, these findings highlight histamine, especially via the H3 receptor, as a promising neuromodulatory target for the comorbidity of OCD and anxiety.

Notably, several H3 receptor agonists, including RAMH and its prodrugs, have entered clinical trials and have proven safe [216]. However, despite this established safety profile and compelling preclinical evidence, H3 receptor‐based therapeutics remain untested in patients with OCD or anxiety disorders. While H3 receptor‐targeting drugs await clinical validation in anxiety disorders, several H1 and H2 receptor antagonists, including hydroxyzine, chlorpheniramine, and promethazine, have been reported to alleviate anxiety symptoms in clinical settings [217, 218, 219, 220]. To bridge this translational gap, developing strategies to target the H3 receptor in the NAc, such as ultrasound‐ or magnetic‐responsive nanoparticle‐based delivery systems for H3 receptor agonists [221, 222], may pave the way for the clinical treatment of OCD (Figure 3). Notably, intranasal delivery of the selective H4 receptor agonist VUF 8430 produces sustained, reversible central effects in freely moving rats [223], establishing a noninvasive precedent for chronically targeting central histamine receptors in vivo. Importantly, while preclinical studies provide a strong mechanistic rationale, this approach remains at the hypothesis‐generating stage. Translating these circuit‐level insights into effective therapies will require addressing significant challenges, including achieving region‐specific drug delivery within interconnected brain circuits and managing potential off‐target effects.

#### 4.2.5 Neuromodulation Therapies

Neuromodulation has attracted increasing attention in the treatment of neuropsychiatric disorders, particularly OCD. The development of invasive and noninvasive procedures, including DBS, tDCS, rTMS, and focused ultrasound, may provide additional options for patients who do not respond to standard treatments (Figure 3).

Among invasive neuromodulation strategies, DBS has demonstrated long‐term efficacy in treatment‐refractory OCD across multiple clinical trials with extended follow‐up periods [224, 225]. Despite its success, the therapeutic mechanisms underlying DBS remain unclear. Multiple different target brain regions have been used for DBS, including the NAc, anterior limb of the internal capsule (ALIC), ventral capsule/ventral striatum, subthalamic nucleus (STN), and inferior thalamic peduncle [226]. Among these targets, stimulation of the STN [227] or NAc [228] has also been reported to relieve anxiety symptoms in clinical trials.

Preclinical studies provide insights into potential mechanisms. Recent work has shown that DBS increases histamine release from histaminergic afferents in the STN, which underlies how STN‐DBS alleviates Parkinsonian motor deficits [229]. While the contribution of histaminergic modulation on STN to OCD remission is still unknown, the beneficial effects of NAc core histaminergic activation in animal models of OCD have already been elucidated [93]. We thus speculate that, similar to STN‐DBS, DBS of the NAc core may also induce histamine release and could subsequently activate presynaptic H3 receptors in the NAc core, which have been reported to inhibit glutamatergic afferent inputs from the prefrontal cortex, and subsequently alleviate OCD and anxiety symptoms. Beyond such local neurochemical actions, ventral striatal high‐frequency stimulation produces target‐specific and state‐dependent modulation of a theta‐responsive distributed subnetwork [230].

Looking forward, the development of personalized, biomarker‐driven closed‐loop DBS systems holds promise for enhancing treatment precision [231]. Preclinical proof‐of‐concept has been demonstrated in animal models. For instance, real‐time closed‐loop optogenetic stimulation of striatal interneurons, triggered by a predictive biomarker (a transient increase in power in the 1–4 Hz band in the OFC), effectively reduced compulsive grooming, with 87% less stimulation time than continuous protocols [232]. While such advances provide important preclinical validation for adaptive stimulation paradigms, significant challenges remain for clinical translation, including the identification of reliable, patient‐specific neurophysiological biomarkers and the development of robust adaptive stimulation algorithms. Computational approaches are likewise beginning to address patient‐specific optimization, as illustrated by 3D point‐cloud learning models that predict individual DBS motor responses from anatomy, electrode geometry, and simulated electric fields [233].

Compared with DBS, techniques such as tDCS and rTMS offer the advantage of noninvasiveness, though their stimulation depth is generally limited to superficial cortical regions. The cortical regions within CSTC circuits may be potential targets for tDCS and rTMS. The dlPFC is currently the most frequent stimulation area in clinical studies of tDCS and rTMS for these conditions [234, 235, 236]. Beyond the dlPFC, low‐frequency rTMS targeting the SMA and add‐on tDCS combined with fluoxetine have also been evaluated in OCD [237, 238]. Whether other cortical regions in the CSTC circuits related to both OCD and anxiety disorders, such as the ACC, vmPFC, dmPFC, and OFC, may be potential therapeutic targets for tDCS and rTMS in OCD treatment, and whether a better therapeutic effect can be achieved, still needs to be further investigated.

Ultrasound‐based neuromodulation encompasses a spectrum of approaches with distinct mechanisms and clinical applications [239]. In its ablative form, high‐intensity focused ultrasound enables precise thermoablative lesioning. Both short‐ and long‐term follow‐up clinical studies show that bilateral thermal lesioning of the ALIC with high‐intensity magnetic resonance‐guided focused ultrasound (MRgFUS) improves the Y‑BOCS and Hamilton Rating Scale for Anxiety (HAM‐A) scores in patients with medically refractory OCD [240, 241, 242]. Clinical studies demonstrate that bilateral thermal lesioning of the ALIC using MRgFUS produces significant and sustained improvements in both OCD and anxiety symptoms in treatment‐refractory patients [242]. A 2025 long‐term study reported a mean Y‑BOCS reduction of approximately 52.3% over 10 years after MRgFUS capsulotomy, supporting its durability as a neurosurgical intervention [243]. Moreover, patients with treatment‐resistant depression experience rapid symptom reduction within a week of bilateral thermal capsulotomy by MRgFUS, which may be attributed to anxiety relief after the procedure [244, 245].

In contrast, low‐intensity focused ultrasound (LIFU) is a noninvasive modality that can reversibly modulate neuronal activity with millimeter‐scale spatial precision and deep tissue penetration [246, 247, 248]. While LIFU has shown promising mood‐enhancing effects in preliminary studies involving chronic pain patients [249] and healthy participants [250], it has not yet been formally evaluated in clinical trials for OCD or anxiety populations. Given its bidirectional neuromodulatory capacity, LIFU holds significant potential as a future strategy for targeting neural substrates underlying OCD–anxiety comorbidity [246, 251]. However, this approach remains primarily experimental, and its therapeutic application for these specific disorders requires further validation. In parallel, advances in super‐resolution ultrasound localization microscopy may help refine target localization and treatment planning for LIFU [252]. Moreover, emerging ultrasound brain–computer interface frameworks may help accelerate the development of transcranial closed‐loop neuromodulation [253].

Finally, exercise represents a distinct, nonpharmacological form of behavioral neuromodulation. Both clinical prospective cohort studies and basic experimental studies have shown that exercise can effectively relieve anxiety [254, 255]. Since OCD is closely related to the basal ganglia, a subcortical motor center, it is likely that precise exercise therapies can also be a potential strategy for the prevention and treatment of comorbid OCD and anxiety. Future research should focus on elucidating the underlying neurobiological mechanisms and optimizing the integration of exercise within comprehensive, multimodal treatment frameworks.

In summary, while first‐line treatments for OCD, SSRIs and CBT, demonstrate efficacy and are believed to modulate shared neural substrates of OCD and anxiety, their therapeutic outcomes remain suboptimal for a substantial proportion of patients. This limitation underscores the need for more precise, mechanism‐based interventions. Emerging strategies that target specific biomarker‐defined dysregulations, such as anomalies in 5‐HT, glutamate, dopamine, and histamine signaling, represent a promising direction. Agents like psilocybin (modulating 5‐HT2A receptors), ketamine, and memantine (targeting NMDARs) have shown preliminary potential in alleviating symptoms of both OCD and anxiety in early reports [182, 183, 198, 201, 202, 203]. However, the current evidence base is largely derived from small‐scale, open‐label studies or case reports, with a notable paucity of large, randomized controlled trials. Moreover, many of these studies lack concurrent, rigorous assessment of anxiety symptoms in OCD populations, leaving the true effect on comorbidity inadequately characterized. Beyond efficacy, the safety and tolerability of these broad neuromodulatory agents, which act on widely distributed neurotransmitter systems, remain significant concerns, necessitating careful risk‐benefit evaluation. A critical future challenge lies in achieving circuit‐ and region‐specificity in intervention, minimizing off‐target effects while correcting the pathological hyperactivity or dysconnectivity within defined networks. In this regard, neuromodulation techniques offer a distinct advantage by enabling anatomically precise targeting. Among these, LIFU represents a particularly promising noninvasive technology capable of modulating deep brain structures with high spatial precision, though its application in psychiatry remains nascent. Furthermore, the development of closed‐loop neuromodulation systems, guided in real‐time by biomarkers of comorbid state, holds immense potential for delivering personalized, adaptive therapy [258]. Nevertheless, these advanced concepts are still largely at the proof‐of‐principle stage. Bridging the translational gap from compelling preclinical mechanisms and pilot clinical observations to validated, safe, and effective personalized treatments for OCD–anxiety comorbidity will require concerted efforts in biomarker discovery, circuit‐level therapeutic targeting, and the conduct of rigorous, pathophysiology‐informed clinical trials.

## 5 Transdiagnostic Implications: Extending the Framework to Other Comorbidities

The extensive exploration of the comorbidity between OCD and anxiety disorders underscores the utility of a multiscale, circuit‐based perspective that transcends traditional diagnostic categories. This approach, however, is not limited to this specific dyad. The high rates of co‐occurrence between OCD and other major psychiatric conditions, most notably MDD and PTSD, compel a broader, transdiagnostic application of the biomarker‐ and circuit‐based framework developed in this review. Examining these comorbidities through this integrated lens not only deepens our understanding of OCD but also tests the robustness and generalizability of the transdiagnostic approach itself.

### 5.1 Shared Circuitry in OCD and MDD

Clinical and meta‐analytic evidence show that nearly half of individuals with OCD experience a lifetime depressive episode, which is associated with greater symptom severity, chronicity, and poorer treatment responses [259, 260]. Notably, clinical studies examining OCD and anxiety disorders have routinely documented significant depressive symptomatology. The efficacy of SSRIs across OCD, anxiety disorders, and MDD supports shared neurochemical underpinnings of these dimensional constructs [151, 152, 261, 262]. Consistent with this, several OCD–anxiety shared susceptibility genes, most notably *SLC6A4* and *MAOA*, are also implicated in MDD, with evidence linking *SLC6A4* promoter methylation and specific *MAOA* polymorphisms to MDD risk (Table 1) [263, 264].

At the circuit level, both disorders exhibit abnormalities within frontolimbic networks, including the amygdala, ACC, and vmPFC, which are critical for emotional regulation and threat processing [132, 265]. Alterations in CSTC circuits, which mediate cognitive control and habit formation, are prominent in OCD and observed in MDD, particularly in relation to rumination and behavioral inertia [265, 266, 267]. Recent neuroimaging studies further indicate that comorbid OCD–MDD is characterized by additional dysfunction in default mode network hubs, such as the mPFC and precuneus, reflecting the superimposition of mood‑related network disturbances onto the core CSTC abnormalities of OCD [268].

These findings provide a translational rationale for stratifying patients with comorbid OCD and MDD based on their predominant circuit dysfunction. For instance, interventions that target the dlPFC to enhance cognitive control and modulate CSTC overactivity, or those that focus on the subgenual ACC to ameliorate primary mood dysregulation, have shown promise in clinical studies for both disorders [235, 236, 269, 270, 271]. Moreover, emerging pharmacological agents investigated in the context of OCD and anxiety, such as psilocybin [262, 272, 273] and ketamine [199, 257], have also demonstrated robust antidepressant effects, underscoring the potential of transdiagnostic treatment strategies that target shared circuit‐level pathology. Together, this integrated perspective advocates for a shift from purely symptom‐based classifications toward mechanism‐based interventions, with the aim of improving outcomes for patients affected by these frequently co‐occurring and clinically intertwined conditions.

### 5.2 Shared Circuitry in OCD and PTSD

Epidemiological data demonstrate a markedly elevated risk of diagnostic co‐occurrence between OCD and PTSD. Among individuals with OCD, approximately 19% meet criteria for lifetime PTSD, whereas among those with PTSD, as many as 41% meet criteria for lifetime OCD [274]. Clinically, this comorbidity profile is associated with more severe OCD symptoms, poorer insight, reduced quality of life, and in some studies diminished treatment response relative to either disorder alone [274].

Genetically, several loci that also appear among OCD–anxiety shared susceptibility genes, notably stress‐axis signaling via *CRHR1*, have been linked to PTSD vulnerability and symptom expression following trauma exposure [275]. In parallel, *MAOA* have been associated with PTSD‐related peripheral epigenetic alterations or symptom‐dimension severity [276].

At a neurocircuitry level, both OCD and PTSD involve disruptions within the fear network, which includes the amygdala, hippocampus, and vmPFC—regions critical for fear expression, extinction learning, and safety signaling [266, 277]. Experimental and neuroimaging studies in OCD demonstrate deficits in fear extinction and recall, paralleled by altered engagement of the vmPFC and amygdala during conditioning tasks, findings that closely resemble those observed in PTSD and point toward a shared impairment in fear‐inhibitory learning [278].

Despite these common circuit‐level disruptions, factor‐analytic and clinical investigations suggest that the symptomatic overlap, particularly in intrusive thoughts and avoidance behaviors, often arises from distinct underlying processes. In PTSD, these symptoms are frequently trauma‐memory‐driven and contextually anchored, whereas in OCD, they are more typically linked to obsession‐driven compulsions and maladaptive habit formation [274]. This distinction calls for a transdiagnostic, circuit‐informed approach to treatment planning. For a given patient, the clinical question becomes whether intrusive and avoidance symptoms are more effectively addressed by targeting trauma memory reconsolidation and impaired extinction within the amygdala–hippocampus–vmPFC network (as in trauma‐focused therapies for PTSD) or by addressing habit‐based compulsive responding and CSTC circuit dysfunction (as in ERP for OCD) [279]. Functional neuroimaging, fear‐learning paradigms, and detailed behavioral phenotyping may help differentiate the predominant mechanism and thus guide the sequencing and selection of interventions tailored to the individual's neurocognitive profile [274].

When viewed through an integrative lens that links genetics, neurocircuitry, and behavior, OCD comorbidity with anxiety disorders, MDD, and PTSD reveals distinct patterns of dysregulation within partially overlapping brain networks. While OCD–anxiety comorbidity is characterized by CSTC hyperactivity and heightened threat appraisal, and OCD–MDD involves more salient disturbances in reward circuits and the default mode network, OCD–PTSD comorbidity centers on the intersection of fear‐network dysfunction with trauma‐related memory and contextual processing. Moving forward, research should focus on deconstructing these comorbid presentations into quantifiable circuit‐based profiles. Such an approach can inform the development of targeted, circuit‐specific interventions, spanning cognitive–behavioral, pharmacological, and neuromodulatory strategies, and pave the way toward a more precise, personalized framework for treating patients with OCD and its major psychiatric comorbidities.

## 6 Conclusion and Future Perspectives

This review has systematically examined the complex relationship between OCD and anxiety disorders through an integrative framework of comorbidity, synthesizing evidence across genetic, neurobiological, and circuit‐level mechanisms. Our analysis establishes that OCD should not be conceptualized as an isolated diagnostic entity but as a component of a broader psychopathological spectrum interconnected at molecular, systems, and clinical levels. Key shared correlates and potential candidate biomarkers—particularly within the CSTC, FPN, and frontolimbic network—underpin the pathophysiology of both conditions. These converging neurobiological substrates highlight the potential of therapeutic strategies that target common pathways, such as glutamatergic, dopaminergic, and histaminergic systems, to provide more effective and integrated treatment for patients with comorbid OCD and anxiety.

Building on these findings, we propose a transdiagnostic, circuit‐based framework for understanding and treating OCD–anxiety comorbidity. This model emphasizes the dynamic, bidirectional interactions between intrusive thoughts, compulsive behaviors, and heightened anxiety, a self‐sustaining, vicious cycle that traps individuals in a persistent pathological loop. While the mechanisms reviewed here contribute to this cycle, the precise neural and psychological processes that initiate and sustain it require further elucidation.

Translating these insights into clinical practice remains challenging. While animal models and emerging therapies provide valuable proofs of concept, a critical field‐wide limitation remains the gap between identifying potential candidate biomarkers and validating them for true clinical utility. Much of the extant evidence, from GWAS correlations to case–control neuroimaging, reflects group‐level associations and lacks the sensitivity, specificity, or prospective predictive value required for individualized care. Consequently, future research must prioritize the prospective validation of these candidates within large, well‐phenotyped, and longitudinal cohorts to transition from correlative frameworks to actionable biomarker‐guided therapy.

Addressing this translational gap also demands moving beyond correlative frameworks to solve a deeper conceptual hurdle: the nature of bidirectional causality. While shared liability is well established, the precise directional influences between OCD and anxiety remain poorly understood. Leveraging shared circuits and genes to develop causal models, via longitudinal tracking and circuit‐specific manipulations, is paramount to determining whether anxiety drives OCD severity or vice versa, thereby informing disease‐modifying interventions.

Ultimately, the next frontier lies in translating causal insights and prospectively validated biomarkers into mechanism‐based personalized medicine. Current approaches to personalization have primarily relied on single‐dimension biomarkers, genetic profiles to predict response to CBT [52, 280], neuroimaging markers to guide neuromodulation targets [269, 281, 282], or symptom subtypes to inform intervention selection [283]. While valuable, these unidimensional approaches are insufficient to capture the complexity of comorbid conditions. The future demands multimodal data integration, synthesizing genetic susceptibility, circuit‐level dysfunction, and detailed clinical phenotyping to construct predictive algorithms for treatment selection. For instance, in a patient with comorbid OCD and anxiety, such an integrated approach could guide a combined therapeutic strategy: (1) selecting pharmacological agents that target specific neurotransmitter systems (e.g., glutamatergic, dopaminergic, or serotonergic modulators) based on genetic markers; (2) identifying anatomical targets for neuromodulation (e.g., specific CSTC, FPN, or frontolimbic network nodes) based on individual circuit dysfunction observed in neuroimaging; and ideally, (3) developing targeted drug delivery systems that enable precise pharmacological intervention in specific brain regions implicated in the comorbidity. In this way, combined biomarker‐driven treatment pathways that integrate pharmacotherapy, neuromodulation, and region‐specific drug delivery can shift the paradigm from sequential trial‐and‐error to mechanism‐based personalization, where multimodal therapeutic choices are anchored in an individual's neurobiological signature.

## Author Contributions

J.N.Z., W.H.Y., F.W., and J.T. conceived of the paper. X.Y.Z., Z.H.L., L.P.S., and B.Q.K. drafted the manuscript and designed the figures. Q.P.Z. and Y.K. refined the manuscript. All authors have read and approved the final paper.

## Funding

This work was supported by STI2030‐Major Projects‐2021ZD0202805 from the Ministry of Science and Technology of China; grants 82522033, 12227808, 32561160133 and 32530049 from the National Natural Science Foundation of China; grant BK20240168 from the Natural Science Foundation of Jiangsu Province, China; grants 492114380005, 492114913211, and 020814380208 from the Fundamental Research Funds for the Central Universities, China; and grant JC2025036 from the Science and Technology Program of Zhenjiang City, China.

## Ethics Statement

The authors have nothing to report.

## Conflicts of Interest

The authors declare no conflicts of interest.

## Data Availability

The authors have nothing to report.
